# Gut microbes metabolize strawberry phytochemicals and mediate their beneficial effects on vascular inflammation

**DOI:** 10.1080/19490976.2024.2446375

**Published:** 2025-01-06

**Authors:** Chrissa Petersen, Adhini Kuppuswamy Satheesh Babu, Ceres Mattos Della Lucia, Henry A. Paz, Lisard Iglesias-Carres, Ying Zhong, Thunder Jalili, J David Symons, Kartik Shankar, Andrew P. Neilson, Umesh D. Wankhade, Pon Velayutham Anandh Babu

**Affiliations:** a Department of Nutrition and Integrative Physiology, College of Health, University of Utah, Salt Lake City, UT, USA; b Arkansas Children’s Nutrition Center, University of Arkansas for Medical Sciences, Little Rock, AR, USA; c Department of Pediatrics, University of Arkansas for Medical Sciences, Little Rock, AR, USA; d Plants for Human Health Institute, Department of Food, Bioprocessing and Nutrition Sciences, North Carolina State University, Kannapolis, NC, USA; e Department of Pediatrics, Section of Nutrition, University of Colorado Anschutz Medical Campus, Aurora, CO, USA

**Keywords:** Diet-derived metabolites, phytochemicals, gut microbiome, vascular, strawberries, host-microbiome interaction

## Abstract

Evidence suggests that a healthy gut microbiome is essential for metabolizing dietary phytochemicals. However, the microbiome’s role in metabolite production and the influence of gut dysbiosis on this process remain unclear. Further, studies on the relationship among gut microbes, metabolites, and biological activities of phytochemicals are limited. We addressed this knowledge gap using strawberry phytochemicals as a model. C57BL/6J mice were fed a standard diet [C]; strawberry-supplemented diet (~2 human servings) [CS]; strawberry-supplemented diet and treated with antibiotics (to deplete gut microbes) [CSA]; high-fat diet (HFD) [HF]; strawberry-supplemented HFD [HS]; and strawberry-supplemented HFD and treated with antibiotics [HSA] for 12 weeks. First, antibiotic treatment suppressed the production of selected metabolites (CSA *vs*. CS), and *p*-coumaric acid was identified as a strawberry-derived microbial metabolite. Second, HFD-induced dysbiosis negatively affected metabolite production (HS *vs*. HF), and hippuric acid was identified as a microbial metabolite in HFD conditions. Third, dietary strawberries improved HFD-induced vascular inflammation (HS *vs*. HF). However, antibiotic treatment reduced metabolite production and abolished the vascular effects of strawberries (HSA *vs*. HS), indicating the importance of gut microbes in mediating the vascular benefits of strawberries *via* metabolites. Fourth, strawberry supplementation decreased *Coprobacillus* that was positively associated with vascular inflammation, whereas it increased *Lachnospiraceae* that was negatively associated with vascular inflammation and positively associated with hippuric acid. Fifth, hippuric acid was negatively associated with vascular inflammation. Our study fills in some pieces of the giant puzzle regarding the influence of gut microbes on the biological activities of phytochemicals. HFD-induced gut dysbiosis negatively impacts metabolite production and a strong association exists among gut microbes, strawberry-derived microbial metabolites, and the vascular benefits of dietary strawberries. Further, our study provides significant proof of concept to warrant future research on the use of strawberries as a nutritional strategy to prevent vascular complications.

## 1. Introduction

The gut microbiome greatly contributes to human health by metabolizing dietary components, producing vitamins, modulating the immune system, and regulating metabolism.^1^ Humans lack digestive enzymes to metabolize dietary components such as soluble fiber and non-nutrient phytochemicals.^1,2^ Gut microbes metabolize the dietary compounds and the dietary compounds support the growth of particular gut microbes, suggesting a symbiotic relationship between diet and gut microbes. Gut bacterial enzymes are efficient in metabolizing dietary phytochemicals, and the resulting metabolites enter the circulation to mediate their biological activities.^2^ Indeed, a healthy gut microbial environment is crucial to get the benefits of dietary phytochemicals through the phytochemical-derived microbial metabolites. However, studies are limited in their ability to identify the roles of gut microbes in mediating the biological activities of phytochemicals through their metabolites and investigating how dietary composition influences the production of these metabolites. In the present study, we addressed this knowledge gap by using dietary strawberries as a model, which is the most consumed berry in the US in addition to blueberries.^3^

Evidence from our lab and others indicates the vascular benefits of dietary strawberries in humans and preclinical models.^4–8^ Strawberries are rich in bioactive polyphenolic compounds such as anthocyanins that consist of an anthocyanidin component (such as cyanidin, delphinidin, malvidin, petunidin, peonidin, or pelargonidin) and a sugar moiety (glucose, arabinose, or galactose).^5^ Strawberries are unique in their composition of anthocyanins compared to other berries, as they are rich in glycosides of pelargonidin.^6^ Studies suggest that ~90% of anthocyanins reach the large intestine and are metabolized by the gut microbes.^9,10^ Therefore, it is likely that the circulating microbial metabolites of anthocyanins could mediate the biological activities as opposed to the parent anthocyanins (which are poorly absorbed) and limited metabolites produced by the host metabolism.^9–11^ However, the contribution of gut microbes to strawberry metabolite production and the influence of dietary composition on this process remains unclear. In addition, the relationship among dietary strawberries, gut microbes, strawberry-derived metabolites, and potential vascular benefits is unknown.

In the present study, we investigated (i) the contribution of gut microbes in producing strawberry flavonoid-derived metabolites, (ii) the impact of diet-induced gut dysbiosis on metabolite production, (iii) the role of gut microbes and strawberry flavonoid-derived microbial metabolites in mediating the vascular benefits of dietary strawberries, and (iv) the relationship among gut microbes, strawberry flavonoid-derived metabolites, and vascular effects of dietary strawberries, using high-fat diet (HFD) mice, an established model for studying vascular complications and gut dysbiosis.

## 2. Materials and methods

### 2.1. Chemicals and reagents

Ampicillin (CAS # 69-52-3), metronidazole (CAS # 443-48-1), neomycin sulfate (CAS # 1405-10-3), and vancomycin (CAS # 1404-93-9) were purchased from Sigma-Aldrich/Millipore (St. Louis, MO, USA). Endothelial Basal Medium (EBM; Catalog # 00190860) was purchased from Lonza (Allendale, NJ, USA). RNAlater (Catalog # 76106), RNeasy Plus mini kit (Catalog # 74136), QuantiTect reverse transcription kit (Catalog # 205313), qPCR SYBR green master mix (Catalog # 204054), and DNeasy PowerLyzer PowerSoil kit (Catalog # 12855–100) were purchased from Qiagen (Valencia, CA, USA). QuantiTect primers glyceraldehyde-3-phosphate dehydrogenase (GAPDH; Catalog # QT01658692), monocyte chemotactic protein-1 (MCP-1; catalog # QT00167832), and interleukin 8 (IL-8; catalog # QT00115647), intercellular adhesion molecule-1 (ICAM1; catalog # QT00155078), vascular cell adhesion molecule-1 (VCAM1; Catalog # QT00128793), and E-Selectin (Catalog # QT00114338) were purchased from Qiagen (Valencia, CA, USA). Phosphatase/protease inhibitor cocktail (Catalog # 78441), fetal bovine serum (FBS; catalog # 10-082-147), calcein-AM (Catalog # C1430), and protein assay kit (Catalog # 23225) were purchased from ThermoFisher Scientific (Waltham, MA, USA). The freeze-dried strawberry powder was provided by the FutureCeuticals (Momence, IL, USA).

### 2.2. Experimental design

The Institutional Animal Care and Use Committee approved the animal experimental protocols at the University of Utah (protocol numbers 18–10005 and 21–09013). C57BL/6J male mice (stock number: 000664) at 6 weeks old were purchased from Jackson Laboratories, USA (Bar Harbor, ME, USA). The mice were housed at the University of Utah Comparative Medicine Center Vivarium under humane conditions. They were housed five per cage with a 12-h light/dark cycle at 23°C ±1°C and 45% ± 5% humidity under artificial light. Mice were acclimated for 1 week before the experiments and fed a rodent diet containing ± high-fat ± freeze-dried strawberry powder and treated with ± an antibiotics cocktail in drinking water for 12 weeks (*n* = 10–15 in each group). The mice were divided into the following groups: mice fed a standard control diet (10% kcal from fat) [C], mice fed a strawberry-supplemented diet [CS], and mice fed a strawberry-supplemented diet and treated with antibiotics cocktail [CSA], mice fed a high-fat diet (HFD; 60% kcal from fat) [HF], mice fed a strawberry-supplemented high-fat diet [HS], and mice fed a strawberry-supplemented high-fat diet and treated with antibiotics cocktail [HSA]. After 12 weeks, mice were anesthetized using 2–5% isoflurane to collect cecum, plasma, and aortic vessels for microbial profiling, analysis of metabolites, and assessment of indices of vascular inflammation, respectively.

Cohort 1: The goal was to determine strawberry flavonoid-derived metabolites and the role of gut microbes in producing these metabolites. Gut microbiome and metabolite data from C, CS, and CSA mice were used to identify strawberry flavonoid-derived microbial metabolites.

Cohort 2: The goal was to determine the effect of HFD-induced dysbiosis on the production of strawberry metabolites. Gut microbiome and metabolite data from HF, HS, and HSA mice were used to determine the impact of HFD-induced dysbiosis on the production of strawberry flavonoid-derived metabolites.

Cohort 3: The goal was to identify the role of gut microbes in producing metabolites and mediating the beneficial effects of dietary strawberries on vascular inflammation, as well as to determine the association among dietary strawberries, gut microbes, strawberry metabolites, and indices of vascular inflammation. Data from C, HF, HS, and HSA mice were used (i) to identify the effect of gut microbes in mediating the vascular effects of dietary strawberries and (ii) to determine the association among gut microbes, strawberry metabolites, and vascular health.

### 2.3. HFD and strawberry-supplemented diet

The freeze-dried strawberry powder was provided by the FutureCeuticals (Momence, IL, USA). The macronutrient composition of freeze-dried strawberry powder was analyzed by Merieux NutriSciences (Crete, IL, USA) (Supplementary Table S1). The customized pelleted modified AIN-93 G diets with ± high-fat ± strawberry supplementation (2.35% freeze-dried strawberry powder in the diet) were prepared by Research Diets Inc (New Brunswick, NJ, USA). The composition of diets is shown in Supplementary Table S2. The fiber types (both soluble and insoluble) and sugar types (glucose, fructose, and sucrose) were matched among these diets. The percentage of strawberry powder in the diet was calculated based on the extrapolation of doses from humans to animals by normalization of the body surface area.^6^ The strawberry dose in the present study is equivalent to the nutritional levels of human consumption (~160 g or 2 cups of fresh strawberries).^5,6^

### 2.4. Antibiotics treatment

Mice in the antibiotic group (CSA and HSA) were provided an antibiotics cocktail [1 g/L ampicillin, 500 mg/L vancomycin, 1 g/L neomycin sulfate, and 1 g/L metronidazole] in drinking water, as we reported recently.^12^ The antibiotic dosage was gradually increased so that the animals adapted to the taste of antibiotics with the depletion of gut microbes without affecting their metabolic health.^12^

### 2.5. Measurement of metabolic parameters

Blood glucose was measured by using a commercial glucometer (Contour Next One). Glucose and insulin tolerance tests were performed as we described previously.^13^ A glucose tolerance test was performed at 12 weeks. Mice were fasted overnight, and glucose was given by intraperitoneal injection (2 g glucose/kg body weight). Blood glucose levels were measured at 0-, 15-, 30-, 60-, and 120-min. An insulin tolerance test was done at 12 weeks following a 4-h fast. Insulin was given by intraperitoneal injection (0.75 U Insulin/kg body weight), and blood glucose levels were measured at 0-, 15-, 30-, 60-, and 120-min.

### 2.6. Assessment of vascular inflammation, NOX and NFκB signaling markers

Terminal experiments were performed at 12 weeks. Mice were anesthetized using 2–5% isoflurane. The chest cavity was opened, and a blood sample was collected via cardiac puncture. The aorta and cecum contents were isolated. The binding of monocytes to the vasculature, inflammatory markers, and NADPH oxidases (NOXs) were assessed as we described previously.^13,14^ Briefly, segments of the abdominal aorta proximal to the iliac bifurcation were used to assess the binding of monocytes to the vascular endothelial layer. Longitudinally opened aortic segments were incubated with EBM-medium containing 1% heat-inactivated FBS for 10 min at 37°C. Then, calcein-AM labeled WEHI78/24 mouse monocytic cells were added to the endothelium-exposed aortic segments. The aortic segments were gently washed with PBS after 30 min of incubation to remove unbound monocytes. The binding of monocytes to aortic segments was visualized, captured, and counted using an Olympus IX73 fluorescence microscope at 40× magnification. The monocytes in five fields of view per aortic segment were used to ensure representative sampling and to calculate the average number of monocytes bound to the aortic vessel. Circulating inflammatory markers such as JE/monocyte chemotactic protein-1 (MCP-1) and KC/interleukin-8 (IL-8) were measured by ELISA kits according to manufacturer’s instructions (R&D Systems, Minneapolis, MN). The mRNA expression of NOXs (NOX2 and NOX4) were measured using RT-PCR. Total RNA was isolated from aortic vessels using RNeasy Plus mini kit (Qiagen, CA). cDNA was synthesized using an RT-PCR kit (Qiagen, CA), and the expression of adhesion molecules was measured with qPCR using SYBR green (Qiagen, CA).

### 2.7. Characterization of plasma metabolites by ultra performance liquid chromatography (UPLC) – mass spectrometry (MS)/MS

Plasma was collected from the unfasted experimental mice after a 12-week treatment for metabolite analysis at two different time points (9 am and 9 pm). These two samples were pooled for each mouse. The extraction and analysis of plasma metabolites were performed as described previously and provided in Supplementary Methods.^15^ As the present study is focused on strawberry flavonoids and their microbial metabolites, a targeted analytical strategy was developed to identify predominant native strawberry flavonoids (anthocyanins and flavan-3-ols) and their likely microbial metabolites based on our pilot study and review of previous literature.^8,16,17^ In this pilot study, C57BL/6J male mice aged 3 months (*n* = 3) were fasted for 10 h and received freeze-dried strawberry powder (500 mg freeze-dried strawberry powder/kg body weight dissolved in sterile water) *via* oral gavage. The plasma samples were collected at different time intervals and pooled for pilot analyses to identify native strawberry flavonoids (anthocyanins and flavan-3-ols) and their microbial metabolites and refine the analytical methods. Based on these steps, the analytical UPLC-MS/MS methods were developed (Supplementary Tables 3 and 4). Supplementary Fig. S1 shows the data from the pilot study. Based on our literature review and the pilot study, a total of 31 likely high-abundance microbial metabolites of strawberry flavonoids were included in the final analytical method (Supplementary Tables 3 and 4). In the present study, metabolites of strawberries were enzymatically deconjugated (to convert sulfate and glucuronide phase-II metabolites back to their native forms) and extracted from plasma by solid-phase extraction using Strata-X plates (Phenomenex, Torrence, CA). Then, strawberry metabolites, which include native compounds, phase-II metabolites, microbial metabolites, and smaller aromatic metabolites, were quantified and characterized by a sensitive high throughput UPLC-MS/MS method on a Waters (Milford, MA) Xevo TQD triple quadrupole instrument in both positive and negative electrospray ionization (ESI) modes. UPLC-MS/MS combines the physical separation capabilities of liquid chromatography with the mass analysis capabilities of mass spectrometry. Data acquisition was carried out by multi-reaction monitoring with parameters established using authentic standards where possible. Data were collected and processed with MassLynx software (version 4.1, Waters). Quantification was based on external standard curves of authentic standards. Compounds for which authentic standards are unavailable were quantified based on external standard curves of similar compounds.^15^

### 2.8. Microbial profiling by high throughput 16S rRNA amplicon sequencing

Microbial profiling was carried out as we described with few modifications.^13,18^ Briefly, bacterial genomic DNA was extracted from experimental animals’ cecum contents. Fifty nanograms of genomic DNA was utilized to amplify the V4 variable region of the 16S rRNA gene using 515F/806 R primers. Forward and reverse primers were dual-indexed and barcoded to accommodate multiplexing 384 samples per run. The library of pooled amplicons was subjected to paired-end sequencing (2 × 250 bp) on an Illumina MiSeq with ~30% PhiX DNA. Processing and quality filtering of reads were performed using scripts in Quantitative Insights into Microbial Ecology 2 (QIIME2) and R packages. The amplicon sequence variant (ASV) approach was used for taxonomic profiling. α-diversity and β-diversity estimation were carried out using QIIME2 v2023.7.^19^

### 2.9. Bioinformatics and data analysis

The Miseq Reporter on the instrument computer automated the demultiplexing, adapter trimming, and generation of fastq files. Subsequent bioinformatics analysis was conducted on the QIIME 2 platform. Initial quality filtering and application of the Deblur algorithm were employed for denoising.^20,21^ Representative amplicon sequence variants (ASVs) were used for phylogenetic tree generation with FastTree, and taxonomy assignment utilized a Naive Bayes classifier trained on the Greengenes 13_8 reference.^22,23^ Sampling depth was evaluated using rarefaction curves in conjunction with the Good’s coverage index. α-Diversity metrics were assessed, including observed genus for richness, Shannon diversity index for diversity, and Pielou’s evenness index for evenness.^24^ β-Diversity was determined through weighted Unifrac distances, and the results were visualized using a principal coordinate analysis (PCoA) plot.

Prism 8.0 (GraphPad, CA, USA) or SPSS Version 25 (IBM) was utilized for statistical analyses and graph generation. Microbial data underwent analysis using the R programming language (version *R*-4.2.2). SPSS was used to perform a one-way analysis of variance (ANOVA) to compare groups at a single time point. When the main effects were significant, Tukey post hoc tests were performed. The data are reported as mean ± SE and p <0.05 was considered significantly different. Differences in plasma metabolites among groups (Cohort 1 and 2) were determined with ANOVA, and where differences were found at a significance level set at p <0.05, the differences were further analyzed post hoc with Fisher’s LSD. The differences in plasma metabolites among groups in Cohort 3 were determined by the homogeneity test of variance (Leven’s test).

Plasma metabolites and relative abundance of microbes at genus-level data from C and CS (for cohort 1) as well as HF and HS (for cohort 2) were used to identify the association among gut microbes and strawberry metabolite production. Plasma metabolites, microbial features (relative abundance of microbes at genus level), and markers of vascular inflammation (monocyte binding to the aortic vessel and circulating inflammatory markers JE/MCP-1 and KC/IL-8) data from C, HF, and HS mice were used to identify the association between the gut microbes, strawberry-flavonoid-derived metabolites, and vascular health (cohort 3). Spearman’s correlations were performed using the Shiny App to determine the association between (i) bacterial abundance data and markers of inflammation, (ii) bacterial abundance data and plasma metabolites, and (iii) plasma metabolites and markers of inflammation. All data are considered statistically significant where *p* < 0.05 and are expressed as mean ± SEM, where appropriate.

## 3. Results

### 3.1. Analytical characterization of strawberry powder

The macronutrient composition analysis indicates that the freeze-dried strawberry powder used in the present study consisted of carbohydrates, proteins, and fat at 73.09%, 7.69%, and 11.31%, respectively (Supplementary Table S1). The insoluble and soluble fibers were 14.33% and 8.26% respectively. Simple sugars such as glucose, fructose, and sucrose were 21.88%, 24.65%, and 1.48%, respectively. This data was used to prepare the freeze-dried strawberry-supplemented diet to match the fiber and sugar contents in all the diets (Supplementary Table S2). We recently reported the phytochemical composition (anthocyanins and other major phytochemicals) of the freeze-dried strawberry powder used in the present study.^18^ The strawberry powder had a high concentration of pelargonidin-3-*O*-glucoside (11.08 mg/g powder), while other phytochemicals such as chlorogenic acid, epicatechin, catechin, quercetin, and kaempferol were found at a lower concentration (<0.8 mg/g dry weight).

### 3.2. Antibiotic treatment efficiently depletes gut microbes

Gut microbial depletion in antibiotics-treated mice was confirmed with a rarefaction plot (Supplementary Fig. S2). Further, PCoA plot indicated the diversity of antibiotics-treated mice is distinct from other groups of mice not treated with antibiotics (Supplementary Fig. S2). Rarefaction curves tended to plateau across treatments, indicating adequate sequencing depth for the characterization of the bacterial community (Supplementary Fig. S2). Moreover, Good’s coverage index showed that at least 98% of the bacterial community was represented across samples. Thus, analyses in this study offer a robust assessment of the cecal bacterial community.

### 3.3. Gut microbes play a pivotal role in the production of strawberry flavonoid-derived metabolites

**Figure 1.**
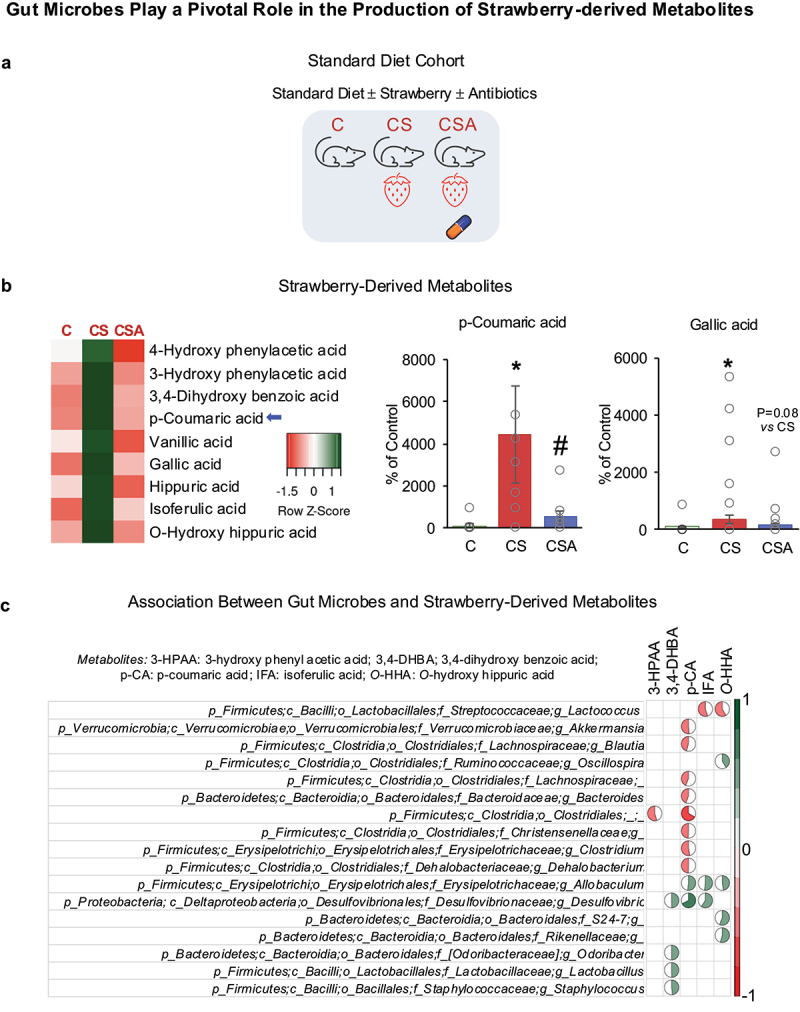
(a) Experimental design for standard diet cohort. (b) Strawberry-derived metabolites in the plasma in standard control diet condition. (c) Spearman’s correlation plot for the association between gut microbes and strawberry-derived metabolites in standard control diet conditions. The pie chart represents the strength of correlation. C: Mice fed a standard control diet; CS: mice fed a strawberry-supplemented diet; CSA: mice fed a strawberry-supplemented diet and treated with an antibiotics cocktail. Values are mean ± SEM (*n* = 10). * *p* < 0.05, CS vs C; and # *p* < 0.05, CSA vs CS.

Data from C, CS, and CSA mice were used to identify the role of gut microbes in the production of strawberry metabolites in standard diet conditions (Figure 1a). Figure 1b shows the metabolites detected in the plasma of experimental mice. Heatmap indicates an increasing trend in several metabolites following the consumption of strawberries (CS vs. C), while antibiotic treatment shows a decreasing trend in many of these metabolites (CSA vs. CS) (Figure 1b). However, some of the changes observed in the circulating metabolites were not significant. We assessed the metabolites in mice fed a physiologically relevant concentration of strawberries. Specifically, the mice in our study were free-living, allowing them to eat whenever they wanted. This natural feeding behavior inherently introduces variability in the timing and quantity of food intake among individual mice, contributing to increased variability in metabolite levels. Hence, although we see a trend in many metabolites, we did not see significant differences in some metabolites due to increased standard deviation. In our study, p-coumaric acid and gallic acid significantly increased in CS vs. C (Figure 1b), indicating the contribution of strawberry phytochemicals in producing these metabolites. A decrease in *p*-coumaric acid and a non-significant decrease (*p* = 0.079) in gallic acid in the antibiotics-treated group indicates the role of gut microbes in producing these metabolites (Figure 1b). Hence, *p*-coumaric acid and gallic acid could possibly be strawberry flavonoid-derived microbial metabolites when strawberries are consumed with a standard diet. In addition, several gut microbes were positively or negatively associated with strawberry-derived metabolites (Figure 1c and Supplementary Fig. S3A).

### 3.4. HFD-induced gut dysbiosis and metabolic changes have a significant impact on the production of strawberry-derived metabolites

**Figure 2.**
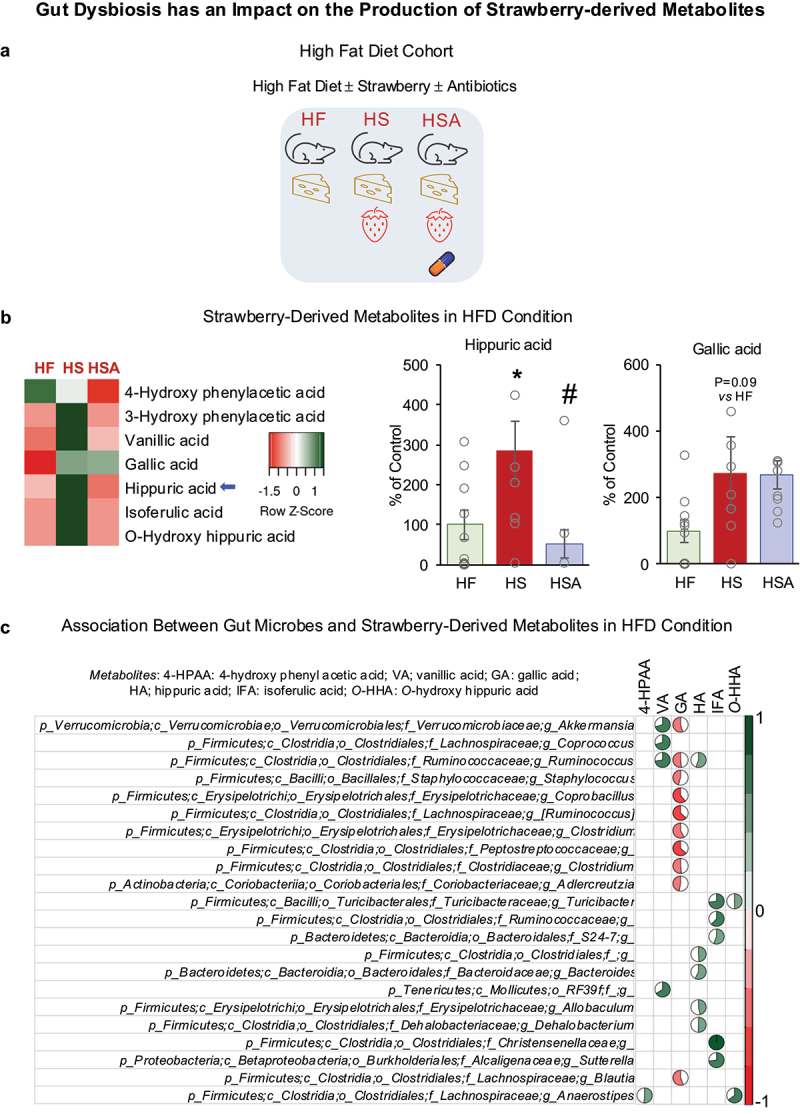
(a) Experimental design for high-fat diet (HFD) cohort. (b) Strawberry-derived metabolites in the plasma in HFD condition. (c) Spearman’s correlation plot for the association between gut microbes and strawberry-derived metabolites in HFD conditions. The pie chart represents the strength of correlation. HF: Mice fed an HFD; HS: mice fed a strawberry-supplemented HFD; HSA: mice fed a strawberry-supplemented HFD and treated with an antibiotics cocktail. Values are mean ± SEM (*n* = 10). * *p* < 0.05, HS vs HF; and # *p* < 0.05, HSA vs HS.

Data from HF, HS, and HSA mice were used to determine the impact of HFD-induced dysbiosis on selected metabolite production (Figure 2a). Figure 2b shows metabolites detected in the plasma of the experimental mice. Heatmap indicates an increasing trend in several metabolites following the consumption of strawberries (HS vs. HF), and antibiotic treatment shows a decreasing trend in many of these metabolites (HSA vs. HS) (Figure 2b). However, not all observed variations are statistically significant. The lack of significance is due to the increased standard deviation, which is influenced by factors previously discussed, including the physiologically relevant concentration of strawberries and the natural feeding behavior of the animals. Hippuric acid was significantly increased, and gallic acid showed a non-significant increase in HS vs HF, indicating the contribution of strawberry phytochemicals in producing these metabolites (Figure 2b). A significant decrease in hippuric acid in the antibiotics-treated group indicates that hippuric acid could be a strawberry-derived microbial metabolite in HFD conditions (Figure 2b). Further, several microbes were positively or negatively associated with strawberry-derived metabolites in HFD condition (Figure 2c and Supplementary Fig. S3B).

### 3.5. Antibiotic treatment abolishes the beneficial effect of dietary strawberries on HFD-induced vascular inflammation

**Figure 3.**
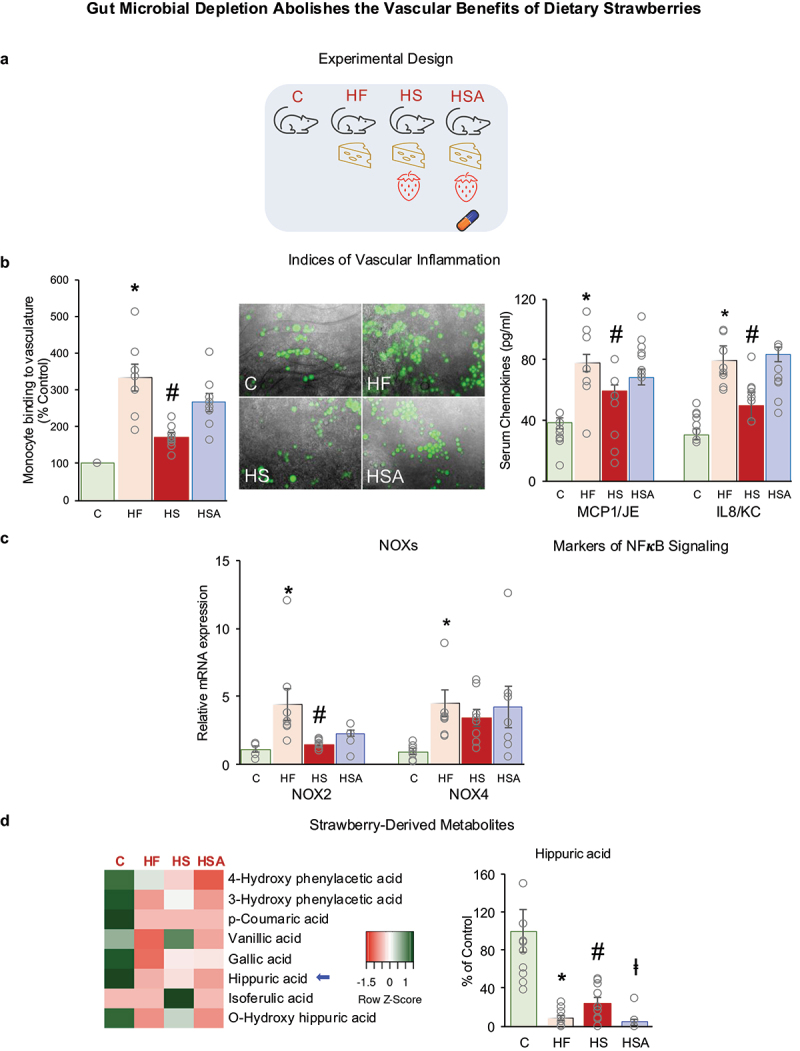
(a) Experimental design. (b) Indices of vascular inflammation and markers of NFκB signaling. (c) NADPH oxidases (NOXs). (d) Strawberry-derived metabolites. c: Mice fed a standard control diet; HF: mice fed an HFD; HS: mice fed a strawberry-supplemented HFD; HSA: mice fed a strawberry-supplemented HFD and treated with an antibiotics cocktail. Values are mean ± SEM (*n* = 7–10). * *p* < 0.05, HF vs C; # *p* < 0.05, HS vs HF; and ⱡ *p* < 0.05, HSA vs HS.

Data from C, HF, HS, and HSA mice were used to determine the role of gut microbes and strawberry-derived microbial metabolites in mediating the vascular benefits of dietary strawberries (Figure 3a). An increased binding of monocytes to aortic endothelium and circulating chemokines (MCP1/JE and KC/IL8) in HF vs C indicates enhanced vascular inflammation, whereas strawberry supplementation suppressed HFD-induced vascular inflammation in HS mice (Figure 3b). However, antibiotic treatment abolished these beneficial effects in HSA mice (Figure 3b). The mRNA expression of NOX2 and NOX4 in the aortic vessel was increased in HF vs C mice. Strawberry supplementation significantly reduced the expression of NOX2 in HS mice but not HSA mice (Figure 3c). Body weight, body fat, and fasting blood glucose were increased in HF vs. C mice, which were not affected by strawberry supplementation and/or antibiotic treatment (Supplementary Table S5). Glucose and insulin tolerance were impaired in HF vs. C mice, which were not improved with strawberry supplementation and/or antibiotic treatment (Supplementary Fig. S4). These metabolic data indicate the direct effect of dietary strawberries on the vasculature.

### 3.6. Hippuric acid is a strawberry-derived microbial metabolite in HFD condition

The heatmap indicated an overall decrease in the selected metabolites pool in HF vs C mice (Figure 3d). Strawberry consumption increased these metabolites (HS vs HF), but a decrease in many of these metabolites was observed with antibiotics treatment (HSA vs HS) (Figure 3d). There was a significant decrease in hippuric acid and gallic acid in HF vs C mice (Figure 3d). Even though there were differences in many of the plasma metabolites with strawberry supplementation as shown in the heatmap, only hippuric acid was statistically different (Figure 3d). Strawberry partially restores hippuric acid production that is lost with HFD (Figure 3d). Strawberry supplementation increased plasma hippuric acid, and antibiotics treatment greatly reduced this phenolic metabolite (Figure 3d), indicating hippuric acid is a strawberry-derived microbial metabolite in HFD condition.

### 3.7. Strawberry supplementation modulates the diversity and composition of gut microbes

**Figure 4.**
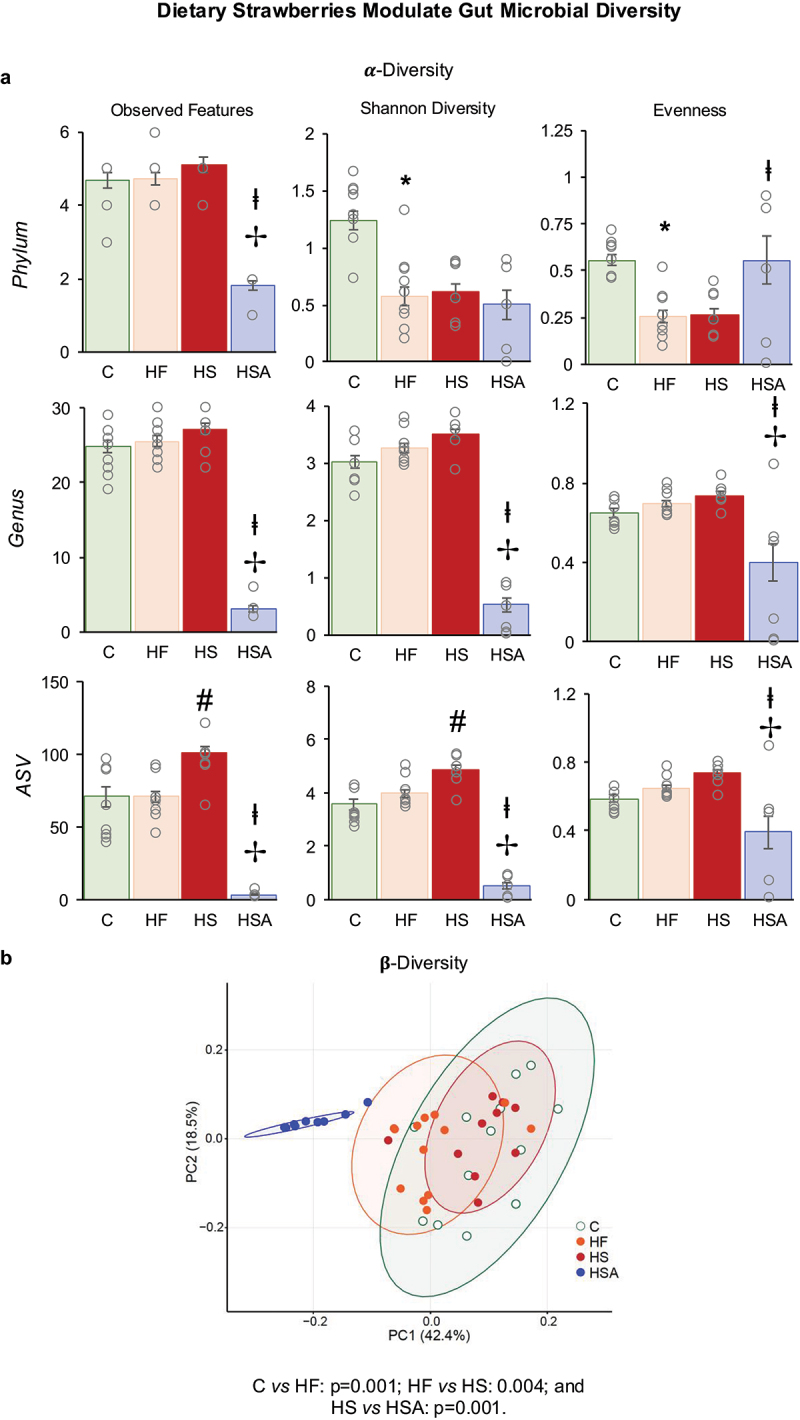
(a) α-Diversity indices of microbial community. (b) β-Diversity of the microbial community. C: Mice fed a standard control diet; HF: mice fed an HFD; HS: mice fed a strawberry-supplemented HFD; HSA: mice fed a strawberry-supplemented HFD and treated with an antibiotics cocktail. Values are mean ± SEM (*n* = 11–14). * *p* < 0.05, HF vs C; # *p* < 0.05, HS vs HF; ✢ *p* < 0.05, HSA vs HF; and ⱡ *p* < 0.05, HSA vs HS.

**Figure 5.**
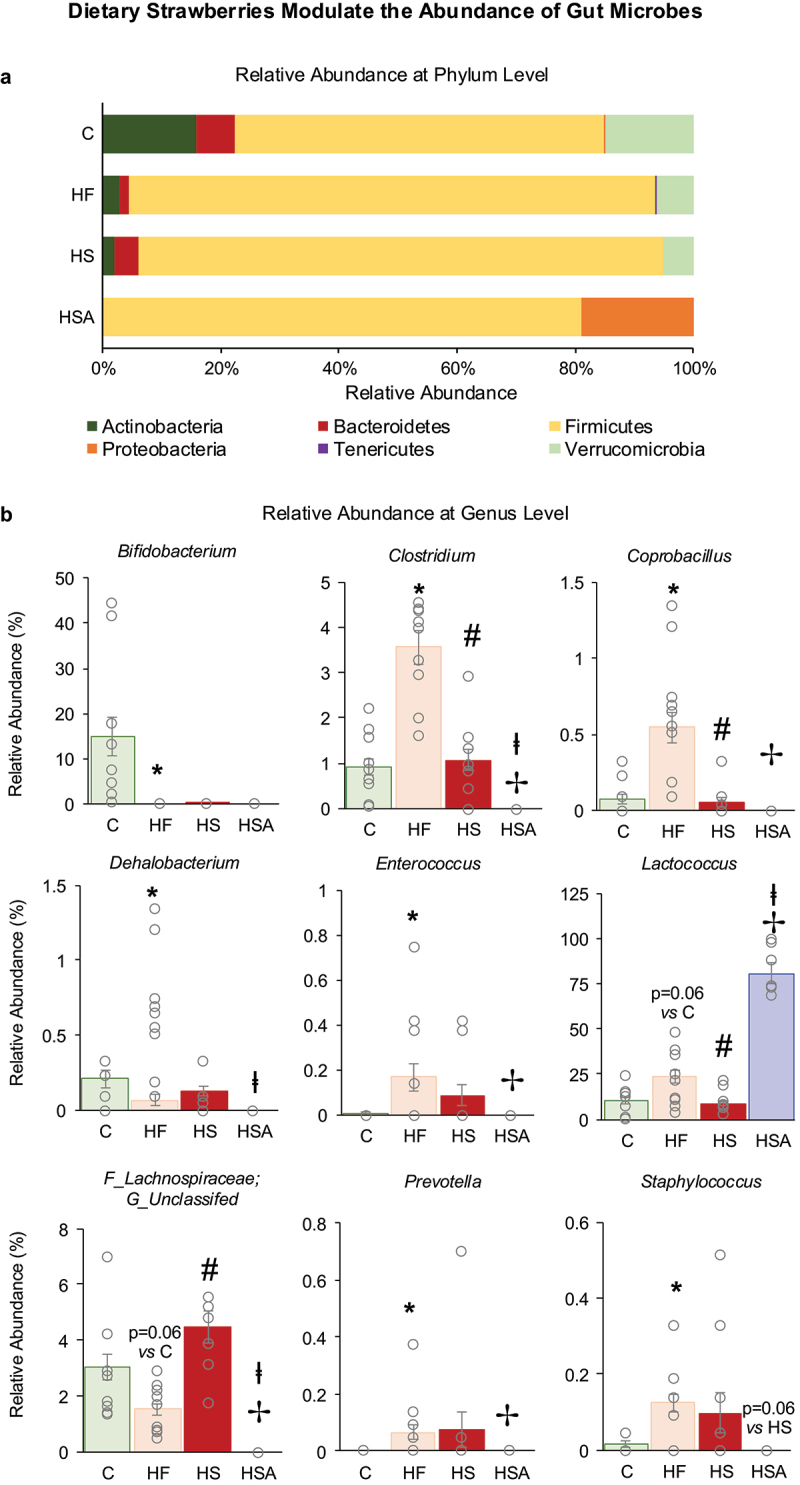
(a) Relative abundance of taxa at the phyla level. (b) Relative abundance of taxa at the genus level. C: Mice fed a standard control diet; HF: Mice fed an HFD diet; HS: mice fed a strawberry-supplemented HFD; HSA: mice fed a strawberry-supplemented HFD diet and treated with an antibiotics cocktail. Values are mean ± SEM (*n* = 11–14). *p < 0.05, HF vs C; #p < 0.05, HS vs HF; ✢p < 0.05, HSA vs HF; and ⱡp < 0.05, HSA vs HS.

α-Diversity was determined by indices such as Observed ASV, Shannon diversity and evenness at different taxonomic levels (phylum, genus and ASV) (Figure 4a). HFD decreased diversity at the phylum level (Shannon diversity and evenness) and strawberry supplementation increased the diversity at the ASV level (Observed ASV and Shannon diversity). Antibiotic treatment greatly decreased the diversity at all three levels (phylum, genus, and ASV). β-diversity is significantly different between the groups (PERMANOVA = 0.001) (Figure 4b). Further, the clusters of C, HF, and HS are distinct as compared to the antibiotics group (HSA). The relative abundance at the phyla level is shown in Figure 5a. Antibiotic treatment eliminated many phyla, including Actinobacteria, Bacteroidetes, Tenericutes, and Verrucomicrobia. A significant alteration in several genera was observed among the experimental groups (Figure 5b). *Clostridium* and *Coprobacillus* were increased with HFD (HF vs C) but decreased with strawberry supplementation (HS vs HF). Unclassified genera belonging to the family *Lachnospiraceae* showed a non-significant decrease (*p* = 0.06) with HFD (HF vs C) but increased with strawberry supplementation (HS vs HF).

### 3.8. Commensal microbes are positively associated with plasma metabolites and negatively associated with indices of vascular inflammation

**Figure 6.**
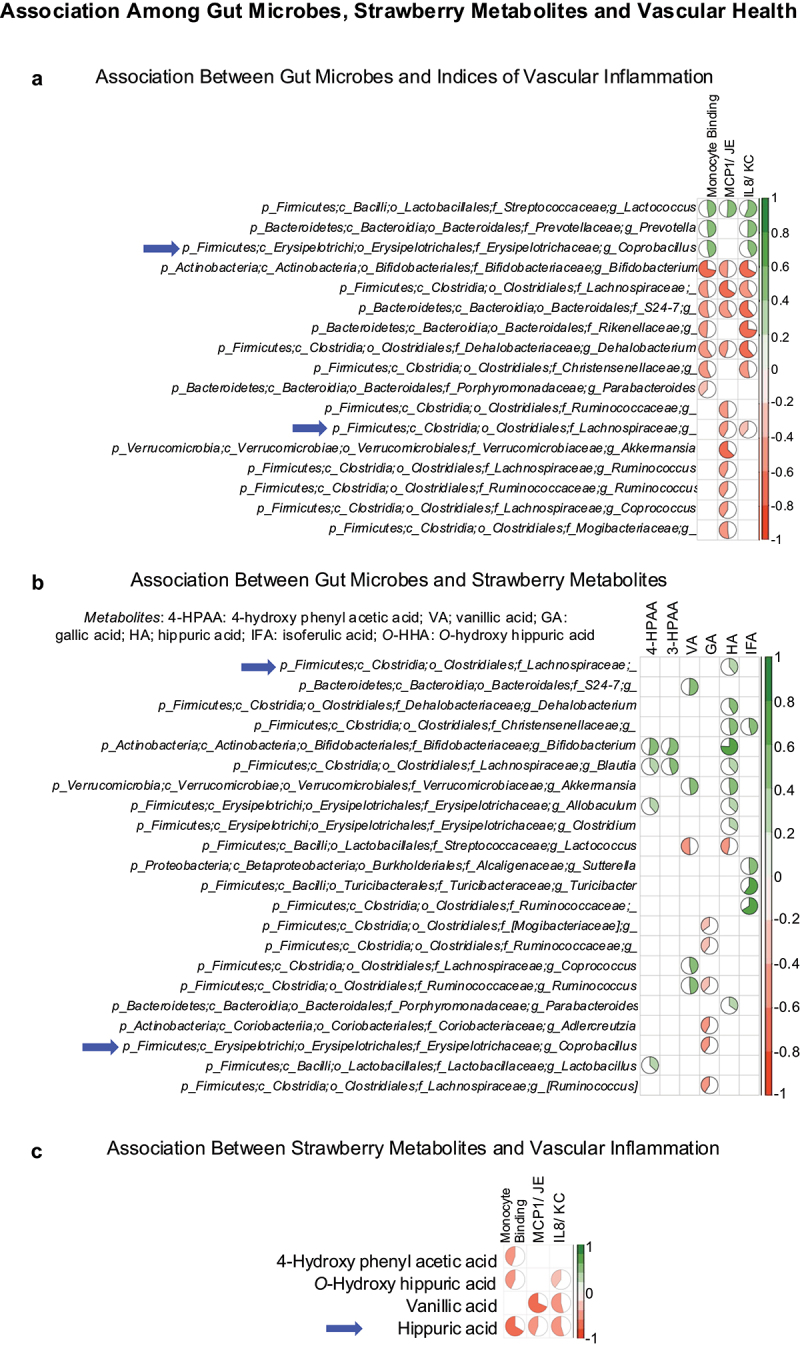
(a) Association between gut microbes and indices of vascular inflammation. (b) Association between gut microbes and strawberry metabolites. (c) Association between strawberry metabolites and indices of vascular inflammation. The pie chart represents the strength of correlation.

Several genera were positively or negatively associated with indices of vascular inflammation (Figure 6a and Supplementary Fig. S3C). Importantly, commensal microbes such as *Akkermansia* and *Bifidobacterium* were negatively associated, whereas an opportunistic microbe *Coprobacillus* was positively associated with indices of vascular inflammation. The selected plasma metabolites were positively or negatively associated with several microbes (Figure 6b and Supplementary Fig. S3D). Specifically, strawberry-derived microbial metabolite hippuric acid was positively associated with several microbes, including commensal microbes *Akkermansia*, *Allobaculum*, *Bifidobacterium* and *Blautia*.

### 3.9. Strawberry-derived microbial metabolite hippuric acid is negatively associated with indices of vascular inflammation

The correlation analysis indicates metabolites such as 4-hydroxy phenylacetic acid, *o*-hydroxy hippuric acid, vanillic acid and hippuric acid were negatively associated with indices of vascular inflammation (Figure 6c and Supplementary Fig. S3E). Importantly, hippuric acid, which was increased with strawberry supplementation, was negatively associated with all the three indices of vascular inflammation (monocyte binding, serum chemokines MCP1/JE and IL8/KC).

### 3.10. Gut microbes, strawberry metabolites and indices of vascular inflammation are closely associated

**Figure 7.**
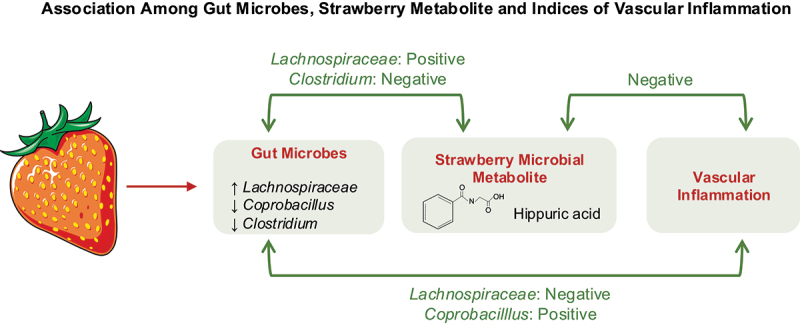
Association among gut microbes, strawberry metabolite, and indices of vascular inflammation.

Gut microbes, strawberry-derived metabolites, and indices of vascular inflammation were closely associated in the present study (Figure 7). For example, *Coprobacillus*, which was increased with HFD and decreased with strawberry supplementation, was positively associated with indices of vascular inflammation, but it was negatively associated with gallic acid. Unclassified genera of the *Lachnospiraceae* family that were decreased with HFD and increased with strawberry supplementation were negatively associated with indices of vascular inflammation but were positively associated with hippuric acid. A strong negative association was observed between hippuric acid and indices of vascular inflammation. These data suggest that gut microbes are essential to producing strawberry-derived metabolites and mediating the vascular beneficial effects of dietary strawberries.

## 4. Discussion

Emerging evidence suggests that a healthy gut microbiome is essential to metabolize dietary phytochemicals. However, significant knowledge gaps exist in understanding the link between phytochemical-derived microbial metabolites and their biological activities. In the present study, we identified strawberry-derived microbial metabolites, determined the impact of dietary composition on the production of metabolites, and assessed the association among gut microbes, strawberry metabolites and vascular effects of dietary strawberries. First, antibiotic treatment suppressed the production of selected strawberry-derived metabolites, and *p*-coumaric acid was identified as a strawberry-derived microbial metabolite. Second, HFD-induced dysbiosis negatively affected metabolite production, and hippuric acid was identified as a strawberry-derived microbial metabolite in HFD conditions. Third, dietary supplementation of strawberries improved HFD-induced vascular inflammation, but antibiotic treatment reduced metabolite production and abolished the beneficial vascular effects of dietary strawberries, indicating the importance of gut microbes in mediating the vascular benefits of strawberries *via* metabolites. Fourth, strawberry supplementation reduced *Coprobacillus* (opportunistic microbe), which is positively associated with vascular inflammation, whereas it increased *Lachnospiraceae* (commensal microbe), which is negatively associated with vascular inflammation and positively associated with hippuric acid. Fifth, hippuric acid was negatively associated with indices of vascular inflammation. Finally, our study indicates a strong relationship among gut microbes, strawberry-derived metabolites, and the vascular benefits of dietary strawberries.

Gut microbes regulate host physiology and pathophysiology through diet-derived microbial metabolites such as trimethylamine, short-chain fatty acids, and phenolic acids.^25^ Trimethylamine N-oxide (derived from trimethylamine) negatively impacts host health, while metabolites such as short-chain fatty acids and phenolic acids provide health benefits to the host.^1^ Gut microbes are crucial to get the benefits of dietary phytochemicals such as berry anthocyanins, as most are poorly absorbed in the small intestine but highly metabolized and absorbed in the large intestine. Studies suggest that only 5–10% of the consumed anthocyanins are absorbed and metabolized through phase I and II metabolism in the small intestine, and the remaining are metabolized in the large intestine by gut microbes.^11,26,27^ Microbial enzymes of species belonging to *Eubacterium*, *Lactobacillus*, and *Bifidobacterium* genera efficiently metabolize dietary anthocyanins into bioactive phenolic acids.^28^ These metabolites enter the circulation and mediate the biological activities of anthocyanins, indicating the effect of dietary anthocyanins on the host mostly depends on gut microbes.^29^ The carbohydrate component of the anthocyanins released during this process provides energy for the microbes, and anthocyanins are considered prebiotics.^6^

Both host and microbial metabolisms contribute to the metabolite production reported in the present study. Evidence suggests that the consumption of berries leads to fluctuations in the pools of small phenolic metabolites already present at baseline.^3^ This is due to the presence of polyphenols in the background diet rather than the appearance of unique berry-derived metabolites, highlighting the widespread nature of polyphenols in the background diet.^3^ Further, due to the presence of compounds, such as *p*-coumaric and ferulic acids, which are both native dietary compounds and can result from microbial metabolism of larger phenolics. The metabolites produced by the microbial metabolism of strawberry phytochemicals were identified by depleting gut microbes with antibiotics. Heatmap analysis indicates an increase in plasma metabolites with strawberry supplementation, whereas antibiotic treatment reduced selected metabolites, confirming the role of gut microbes in producing these metabolites. The standard diet cohort indicates that gut microbes are vital in producing *p*-coumaric and gallic acid. Further, dietary composition and diet-induced gut dysbiosis negatively impact metabolite production. Consistent with the standard diet cohort, strawberry supplementation increased circulating metabolites in the HFD cohort and was reduced with antibiotics treatment. In the present study, a 3-fold increase in hippuric acid with strawberry supplementation that reduced back to baseline with antibiotic treatment in the HFD conditions indicates the pivotal role of gut microbes in producing hippuric acid from strawberry phytochemicals. It is well established from our lab and others that a HFD significantly influences gut microbes.^6,30^ The alteration in the gut microbiome (gut dysbiosis) in the HFD-fed mice could explain the differences in the metabolite production observed in the standard diet and HFD cohorts. This is further confirmed by the differences observed in the association between metabolites and gut microbes in standard diet and HFD conditions. Studies indicate that in addition to the food matrix (higher fat content), the animals’ pathological condition affects the metabolism of phytochemicals and the production of microbial metabolites.^31^ Our study indicates that one-size-fits-all for food’s bioactive components is inaccurate. The metabolism by microbes varies significantly with the dietary composition and health status of the host, suggesting that other aspects of diet and gut microbial communities affect the disease mitigation properties of fruits and vegetables. Studies to understand these interactions and ways to improve overall diet are crucial for those who would benefit from their diet.

Vascular inflammation, characterized by the binding of monocytes to the aortic endothelium and their subsequent transmigration into the subendothelial space prominently contributes to the development of vascular diseases such as atherosclerosis.^6^ Our recent studies demonstrated the beneficial vascular effects of dietary strawberries.^5,6^ In the present study, dietary supplementation of strawberries reduced HFD-induced vascular inflammation, as shown by a decrease in the monocyte binding to the vasculature and circulating chemokines. However, subsequent antibiotic treatment failed to improve vascular inflammation and abolished the beneficial vascular effects of dietary strawberries. Evidence indicates vascular inflammation is mediated through NADPH oxidase 2 (NOX2) activated NFκB signaling.^5^ NFκB activation upregulates genes associated with vascular complications, such as MCP1/JE and IL8/KC.^5,14^ We have previously shown that dietary strawberries reduce NOX2 expression in diabetic mice.^5^ In the present study, HFD increased the expression of NOX2 and NOX4, which was associated with an increase in the circulating chemokines MCP1/JE and IL8/KC. Strawberry supplementation reduced NOX2 expression and circulating chemokines, but antibiotics treatment failed to exert such an effect. Further, reduced circulating metabolites were observed in mice fed HFD compared to the standard diet, possibly due to the alteration in the gut microbes. These metabolites are produced by many dietary sources, including soluble fiber. Dietary supplementation of strawberries increases the pool of these phenolic metabolites but is reduced with subsequent antibiotic treatment. Notably, the highest levels of hippuric acid were in the standard diet-fed mice, which was significantly lowered with HFD but partly restored with strawberry supplementation. Antibiotic treatment significantly reduced hippuric acid. Strawberry supplementation increases hippuric acid but is reduced with antibiotic treatment, indicating that gut microbes are essential to mediate the biological effects of dietary strawberries via microbial metabolites.

Microbial profiling indicates a significant difference in β-diversity among the experimental groups, indicating the influence of HFD, strawberries, and antibiotics on the gut microbiome. The relative abundance at the genus level indicates an increase in *Clostridium*, *Coprobacillus*, *Dehalobacterium*, *Enterococcus*, *Prevotella*, and *Staphylococcus* in HFD-fed mice with a decrease in *Bifidobacterium* and a decreasing trend in unclassified genus belongs to the family *Lachnospiraceae*. Strawberry supplementation reduced the relative abundance of *Clostridium* and *Coprobacillus* while increasing the *Lachnospiraceae* unclassified genus. In the present study, the unclassified genus of the *Lachnospiraceae* family was negatively associated with indices of vascular inflammation (monocyte binding, MCP1/JE, and IL8/KC) and positively associated with strawberry-derived metabolite hippuric acid. *Lachnospiraceae* are butyrate producers through the fermentation of dietary fiber and exhibit anti-inflammatory properties.^32^ Human studies indicate a decreased abundance of *Lachnospiraceae* in vascular complications such as hypertension and coronary artery disease.^33,34^ *Lachnospiraceae* was shown to possess anti-atherosclerotic effects by reducing gut metabolites trimethylamine N-oxide, which is linked to atherosclerosis.^35^ A lifestyle-based immersion program (100% plant-based food, stress management, and exercise) was shown to increase *Lachnospiraceae*, which is associated with a reduction in cardiovascular risk factors (blood pressure, cholesterol, TMAO, and c-reactive protein).^36^ These studies suggest *Lachnospiraceae* is a commensal microbe. In the present study, a decreased trend was observed for *Lachnospiraceae* in HFD-fed mice, but strawberry supplementation increased their abundance. Thus, there is a possibility that strawberries exert beneficial vascular effects through *Lachnospiraceae*, which exhibits a negative association with vascular inflammation.

*Coprobacillus* was positively associated with vascular inflammation. Human studies indicate an increased abundance of *Coprobacillus* in vascular complications such as hypertension and with consumption of a Western diet.^37,38^ *Clostridium* species were shown to be positively associated with ICAM-1 and selectins that contribute to vascular inflammation.^39^ Patients with hypertension exhibit a higher abundance of *Clostridium*.^34^ Germ-free mice colonized with fecal material from coronary artery disease patients had an increased abundance of *Clostridium* associated with vascular stiffness.^40^ These studies suggest the opportunistic properties of *Coprobacillus* and *Clostridium*. Strawberry supplementation reduced these two genera, suggesting this could be one of the possible reasons for the beneficial effects of dietary strawberries on the vasculature. The reported effects of dietary strawberries on vascular inflammation and gut microbes are mostly mediated by the strawberry phytochemicals and are unlikely to be mediated by fiber, as the diets used in our study were matched for soluble and insoluble fibers.

Hippuric acid is a gut-derived metabolite produced from dietary phenolics or endogenously from phenylalanine.^41^ The microbial breakdown of phenolic compounds produces benzoic acid, which is converted into hippuric acid through conjugation with a glycine moiety in the liver.^42^ Hence, the synthesis of hippuric acid mainly depends on gut microbes. *Clostridium* species play a crucial role in the production of hippuric acid.^43^ Interestingly, *Clostridium* is positively associated with hippuric acid in the present study. Human studies indicate that hippuric acid is one of the most abundant metabolites in circulation following the intake of dietary strawberries.^3^ Though strawberry increases hippuric acid, it is a nonspecific marker as other dietary phenolics could produce it. Consumption of berry anthocyanins has been shown to enhance flow-mediated dilation accompanied by elevated plasma concentrations of hippuric acid in healthy humans.^44^ Dietary berries increased the plasma hippuric acid by 6–7-fold, and hippuric acid was favorably associated with lipid profile.^45^ Hippuric acid at a physiologically relevant dosage (at µM concentration) was shown to inhibit monocyte binding to the vascular endothelial cells.^46^ Further, hippuric acid has exhibited cardiovascular benefits via anti-platelet activity.^47^ These studies suggest the cardiovascular benefits of hippuric acid. However, hippuric acid at mM concentrations (1–4 mM) reportedly impairs endothelial function in human aortic endothelial cells, as shown by decreased expression of eNOS, enhanced reactive oxygen species, and increased expression of adhesion molecule ICAM1 in human aortic endothelial cells.^48^ This could possibly be due to the supraphysiological concentrations of hippuric acid (mM) used in this study. In the present study, hippuric acid was positively associated with commensal and negatively associated with opportunistic microbes. Further, hippuric acid was negatively associated with indices of vascular inflammation. Hence, hippuric acid could potentially contribute to the vascular benefits of dietary strawberries, as reported in the present study. Nevertheless, it is crucial to acknowledge that the metabolites pool includes other compounds, and their cumulative effects should be considered as additional factors influencing the outcomes.

Our results should be interpreted in the context of the following limitations. (1) Our study focused on flavonoids from strawberries and their associated gut microbial metabolites. We did not quantify plasma levels of native ellagic acid from strawberries nor its known microbial metabolites (urolithins).^16^ These compounds may be present in the circulation after strawberry consumption and could modulate vascular function. Our future studies will focus on the targeted characterization of ellagic acid and untargeted phenolic metabolites. (2) We did not administer specific gut microbes or use stable isotope tracing with labeled phytochemicals to identify the microbes or microbial genes responsible for producing strawberry-derived metabolites. Future studies are warranted using stable isotope tracing and administering specific gut microbes. Despite these limitations, our study is significant in the field due to several key strengths. (1) Our study showed the importance of gut microbes in metabolizing dietary phytochemicals and mediating their biological effects. (2) It demonstrated the impact of HFD-induced gut dysbiosis on metabolite production. (3) It established the association among gut microbes, strawberry-derived metabolites, and vascular health at the physiologically relevant concentration of dietary strawberries.

## 5. Conclusions

We found a strong association between dietary strawberries, gut microbes, and strawberry-derived metabolites and improved vascular health. Data from the present study fill in some pieces of the giant puzzle regarding the influence of strawberry intake on the gut-vasculature axis. Further, our study provides significant proof of concept to warrant future research on the use of dietary strawberries as a strategy to prevent vascular complications and the mechanisms by which their metabolites exert these benefits.

## Supplementary Material

4_Supplementary_Materials.docx

## Data sharing statement

The sequencing data sets are being submitted to the Sequence Read Archive repository [NIH’s primary archive of high-throughput sequencing data] with the accession number PRJNA1079001. All other relevant data are provided in the Supplementary Information section.
