# Supplementary material for: Gut microbes metabolize strawberry phytochemicals and mediate their beneficial effects on vascular inflammation
Source: Gut Microbes. 2025 Jan 6;17(1):2446375. doi: 10.1080/19490976.2024.2446375 (PMC12931721; doi:10.1080/19490976.2024.2446375)
Supplement: 4_Supplementary_Materials.docx [file KGMI_A_2446375_SM0555.docx]

**SUPPLEMENTARY MATERIALS**

**Gut Microbes Metabolize Strawberry Phytochemicals and Mediate Their Beneficial Effects on Vascular Inflammation**

Chrissa Petersen^1^, Adhini Kuppuswamy Satheesh Babu^1^, Ceres Mattos Della Lucia^1^, Henry A. Paz^2,3^, Lisard Iglesias-Carres^4^, Ying Zhong^2,3^, Thunder Jalili^1^, J David Symons^1^, Kartik Shankar^5^, Andrew P. Neilson^4^, Umesh D. Wankhade^2,3^, Pon Velayutham Anandh Babu^1^

*^1^Department of Nutrition and Integrative Physiology, College of Health, University of Utah, Salt Lake City, Utah 84112, USA. ^2^Arkansas Children’s Nutrition Center, and the ^3^Department of Pediatrics, University of Arkansas for Medical Sciences, Little Rock, AR 72205, USA. ^4^Plants for Human Health Institute, Department of Food, Bioprocessing and Nutrition Sciences, North Carolina State University, Kannapolis, NC 28081, USA. ^5^Department of Pediatrics, Section of Nutrition, University of Colorado Anschutz Medical Campus, Aurora, CO 80045, USA.*

Running title: Diet-Derived Microbial Metabolites

**SUPPLEMENTARY METHODS & TABLES**

**Table 1.** Macronutrient composition of freeze-dried strawberry powder

| **Macronutrient** | **% w/w** |
| --- | --- |
| Protein | 7.69 |
| Fat | 11.31 |
| Carbohydrate | 73.09 |
| Total Dietary Fiber | 22.59 |
| Insoluble Dietary Fiber | 14.33 |
| Soluble Dietary Fiber | 8.26 |
| Sugars |  |
| Fructose | 24.65 |
| Glucose | 21.88 |
| Sucrose | 1.48 |
| Maltose | <0.25 |
| Lactose | <0.25 |

**Table 2.** Rodent diets with 10 or 60 kcal% fat and supplemented with ± 2.35% freeze-dried strawberry powder.

|  | Diet with  10 kcal% fat | Diet with  10 kcal% fat supplemented with strawberry powder | Diet with  60 kcal% fat | Diet with  60 kcal% fat supplemented with strawberry powder |
| --- | --- | --- | --- | --- |
| **Ingredient** | *g* | *g* | *g* | *g* |
| Casein | 200 | 198.1 | 200 | 198.6 |
| L-Cystine | 3 | 3 | 3 | 3 |
|  |  |  |  |  |
| Corn Starch | 506.2 | 506.2 | 0 | 0 |
| Maltodextrin 10 | 112.1 | 111 | 112.1 | 111.4 |
| Sucrose | 68.8 | 67.5 | 68.8 | 67.8 |
| Fructose | 6.8 | 0 | 6.8 | 1.8 |
| Dextrose | 5.6 | 0 | 5.6 | 1.5 |
|  |  |  |  |  |
| Cellulose, BW200 | 48.7 | 44.5 | 48.7 | 45.6 |
| Inulin | 1.3 | 0 | 1.3 | 0.3 |
|  |  |  |  |  |
| Corn Oil | 25 | 25 | 25 | 25 |
| Lard | 20 | 19.2 | 245 | 244.4 |
|  |  |  |  |  |
| Mineral Mix S10026 | 10 | 10 | 10 | 10 |
| DiCalcium Phosphate | 13 | 13 | 13 | 13 |
| Calcium Carbonate | 5.5 | 5.5 | 5.5 | 5.5 |
| Potassium Citrate, 1 H2O | 16.5 | 16.5 | 16.5 | 16.5 |
|  |  |  |  |  |
| Vitamin Mix V10001 | 10 | 10 | 10 | 10 |
| Choline Bitartrate | 2 | 2 | 2 | 2 |
|  |  |  |  |  |
| Freeze Dried Strawberry Powder | 0 | 24.83 | 0 | 18.20 |
|  |  |  |  |  |
| Total | 1054.5 | 1056.33 | 773.3 | 774.6 |
| **Freeze Dried Strawberry Powder (%)** | **0** | **2.35** | **0** | **2.35** |
|  |  |  |  |  |
|  | Diet with  10 kcal% fat | Diet with  10 kcal% fat supplemented with strawberry powder | Diet with  60 kcal% fat | Diet with  60 kcal% fat supplemented with strawberry powder |
| **Macronutrients** | *g* | *g* | *g* | *g* |
| Protein | 203 | 203 | 203 | 203 |
| Carbohydrate | 710 | 710 | 203.8 | 203.8 |
| Fat | 45 | 45 | 270 | 270 |
| Soluble Fiber | 1.3 | 1.3 | 1.3 | 1.3 |
| Insoluble Fiber | 48.7 | 48.7 | 48.7 | 48.7 |
| Sucrose | 68.8 | 68.8 | 68.8 | 68.8 |
| Fructose | 6.8 | 6.8 | 6.8 | 6.8 |
| Dextrose | 5.6 | 5.6 | 5.6 | 5.6 |
| **Macronutrients** | *kcal%* | *kcal%* | *kcal%* | *kcal%* |
| Protein | 20.0 | 20.0 | 20.0 | 20.0 |
| Carbohydrate | 70.0 | 70.0 | 20.1 | 20.1 |
| Fat | 10.0 | 10.0 | 59.9 | 59.9 |
| Total | 100.0 | 100.0 | 100.0 | 100.0 |

**LC-MS/MS Method for detection of strawberry phenolic metabolites**

***Instrument***

Waters Acquity I-class UPLC coupled to a XEVO TQD triple quadrupole mass spectrometer

***Column***

Pre-column: Waters ACQUITY UPLC BEH C18; 1.7 um (2.1 x 50 mm)

Analytical column: Waters ACQUITY UPLC BEH C18; 1.7 um (2.1 x 5 mm)

**Negative mode analysis**

***Mobile phases***

A: 0.1 % Formic acid (FA) in acetonitrile (ACN)

B: 0.1 % FA in water

***Gradient***

| **Time (min)** | **Flow (mL/min)** | **A (%)** | **B (%)** | **Curve** |
| --- | --- | --- | --- | --- |
| 0 | 0.5 | 0 | 100 | 6 |
| 0.5 | 0.5 | 6 | 94 | 6 |
| 2 | 0.5 | 9 | 91 | 6 |
| 3 | 0.5 | 13 | 87 | 6 |
| 4.5 | 0.5 | 35 | 65 | 6 |
| 5.2 | 0.5 | 0 | 100 | 6 |
| 6 | 0.5 | 0 | 100 | 6 |

***Other settings***

| Sampler T(°C) | 7 |
| --- | --- |
| Source T(°C) | 150 |
| Dessolvation T(°C) | 350 |
| Capillary voltage (kV) | 2.5 |
| Conce voltage (V) | 41 |
| Desolvation nitrogen gas flow (L/h) | 720 |
| Cone nitrogen gas flow (L/h) | 5 |
| Electrospray ionization mode | Negative |

**Table 3.** Negative Mode Analysis

| **Multi-reaction monitoring (MRM) table (negative mode)** | | | | | | | | |
| --- | --- | --- | --- | --- | --- | --- | --- | --- |
| **CAS** | **Compound** | **MW** | **Formula** | **Retention Time (Minute)** | **Transition Window**  **(Time)** | **MS/MS Transition (m/z)** | **Cone Voltage (V)** | **Collision Energy (eV)** |
| 99-06-9 | 3-Hydorxybenzoic acid | 138.12 | C7H6O3 | 1.91 | 1.1-2.2 | 136.87>92.96 | 30 | 12 |
| 99-96-7 | 4-Hydroxybenzoic acid | 138.12 | C7H6O3 | 1.37 | 1.1-2.6 | 136.87>92.96 | 30 | 12 |
| 69-72-7 | Salicylic acid (2-Hydroxybenzoic acid) | 138.12 | C7H6O3 | 3.75 | 3.4-4.1 | 137.13>65.00 | 32 | 24 |
| 621-37-4 | 3-Hydroxyphenylacetic acid | 152.15 | C8H8O3 | 2.03 | 1.4-2.4 | 151.01>107.00 | 30 | 5 |
| 156-38-7 | 4-Hydroxyphenylacetic acid | 152.15 | C8H8O3 | 1.67 | 1.4-2.4 | 151.05>107.40 | 28 | 18 |
| 99-50-3 | 3,4-Dihydroxybenzoic acid | 154.12 | C7H6O4 | 0.95 | 0.2-2.5 | 152.95>108.99 | 28 | 14 |
| 490-79-9 | 2,5-dihydroxybenzoic acid | 154.12 | C7H6O4 | 1.41 | 0.2-2.5 | 152.95>108.99 | 28 | 14 |
| 89-86-1 | 2,4-Dihydroxybenzoic acid | 154.12 | C7H6O4 | 1.67 | 0.2-2.5 | 152.95>108.99 | 28 | 14 |
| 501-98-4 | p-Coumaric acid | 164.16 | C9H8O3 | 2.65 | 2.3-3 | 163.00>119.10 | 30 | 16 |
| 614-60-8 | o-Coumaric acid | 164.16 | C9H8O3 | 3.8 | 3.5-4.1 | 163.14>119.47 | 26 | 18 |
| 621-54-5 | 3-Hydroxyophenylpropionic | 166.17 | C9H10O3 | 3.05 | 2.6-3.5 | 165.07>120.70 | 24 | 16 |
| 121-34-6 | Vanillic acid | 168.15 | C8H8O4 | 1.79 | 1.5-2.3 | 166.98>151.98 | 28 | 12 |
| 645-08-9 | Isovanillic | 168.15 | C8H8O4 | 2.06 | 1.5-2.3 | 167.14>107.98 | 28 | 18 |
| 149-91-7 | Gallic acid | 170.12 | C7H6O5 | 0.66 | 0.4-1.1 | 169.02>125.00 | 34 | 16 |
| 495-69-2 | Hippuric Acid | 179.17 | C9H9NO3 | 1.75 | 1.5-2.1 | 178.01>76.98 | 28 | 16 |
| 331-39-5 | Caffeic acid | 180.16 | C9H8O4 | 1.81 | 1.5-2.1 | 179.00>135.10 | 32 | 22 |
| 306-08-1 | Homovanillic acid | 182.17 | C9H10O4 | 2.15 | 1..8-2.7 | 181.22>122.01 | 20 | 12 |
| 1078-61-1 | 3,4-Dihydroxyphenylpropionic acid | 182.17 | C9H10O4 | 1.72 | 1.3-2.1 | 181.14>59.00 | 26 | 18 |
| 99-24-1 | Methylgallate | 184.15 | C8H8O5 | 1.56 | 1.2-1.9 | 183.07>123.97 | 40 | 20 |
| 77-95-2 | Quinic acid | 192.17 | C7H12O6 | 0.34 | 0.2-0.8 | 191.16>85.01 | 38 | 26 |
| 1135-24-6 | Ferulic acid | 194.178 | C10H10O4 | 3.23 | 3.0-3.9 | 193.22>134.00 | 28 | 16 |
| 537-76-5 | Isoferulic acid | 194.178 | C10H10O4 | 3.49 | 3.0-3.9 | 193.22>134.00 | 28 | 16 |
| 1637-75-8 | 3-Hydroxyhippuric acid | 195.17 | C9H5NO4 | 1.14 | 0.9-1.4 | 194.01>92.92 | 32 | 22 |
| 487-54-7 | o-Hydroxyhippuric acid | 195.17 | C9H5NO4 | 2.69 | 2.4-3.1 | 194.15>93.01 | 20 | 22 |
| 1135-15-5 | 3-Hydroxy-4-methoxyphenylpropionic acid | 196.2 | C10H12O4 | 3.43 | 3.1-3.7 | 194.98>136.02 | 32 | 12 |
| 56355-43-2 | 4-Hydroxy-3-methoxyphenylpropionic acid | 196.2 | C10H12O4 | 3.07 | 2.7-3.6 | 195.17>136.07 | 28 | 14 |
| 831-61-8 | Ethyl gallate | 198.17 | C9H10O5 | 2.88 | 2.6-3.25 | 197.01>123.90 | 30 | 30 |
| 530-57-4 | 4-Hydroxy-3,5-dimethoxybenzoic acid (Syringic acid) | 198.17 | C9H10O5 | 2.1 | 1.8-2.3 | 197.07>182.01 | 36 | 14 |
| 2316-26-9 | 3,4-Dimethoxycinnamic acid | 208.21 | C11H12O4 | 4.14 | 3.8-4.3 | 207.17>102.96 | 30 | 12 |
| 4385-56-2 | 3,5-Dimethoxy-4-hydroxyphenylacetic acid | 212.2 | C10H12O5 | 2.5 | 2.2-2.7 | 211.16>152.07 | 26 | 12 |
| 530-59-6 | Sinapic acid | 224.21 | C11H12O5 | 3.39 | 3.1-3.6 | 223.16>208.03 | 28 | 12 |
| 520-18-3 | Kaempferol | 286.24 | C15H10O6 | 4.68 | 4.5-5.2 | 285.02>150.07 | 58 | 18 |
| 154-23-4 | Catechin | 290.27 | C15H16O6 | 1.54 | 1.2-2.7 | 289.10>109.00 | 33 | 24 |
| 490-46-0 | Epicatechin | 290.27 | C15H16O6 | 2.33 | 1.2-2.7 | 289.10>109.00 | 33 | 24 |
| 117-39-5 | Quercetin | 302.23 | C15H10O7 | 4.34 | 4.0-5 | 300.98>150.96 | 38 | 20 |
| 327-97-9 | Chlorogenic acid | 354.31 | C16H18O9 | 1.66 | 0.9-2.6 | 353.02>190.97 | 26 | 18 |
| 480-18-2 | Taxifolin | 304.35 | C15H12O7 | 3.25 | 3.0-4.0 | 303.2>124.99 | 30 | 22 |

**Positive mode analysis**

***Mobile phases***

A: 0.1 % Formic acid (FA) in acetonitrile (ACN)

B: 2 % FA in water

***Gradient***

| **Time (min)** | **Flow (mL/min)** | **A (%)** | **B (%)** | **Curve** |
| --- | --- | --- | --- | --- |
| 0 | 0.5 | 0 | 100 | 6 |
| 0.5 | 0.5 | 6 | 94 | 6 |
| 2.66 | 0.5 | 9 | 91 | 6 |
| 4.33 | 0.5 | 13 | 87 | 6 |
| 6.5 | 0.5 | 35 | 65 | 6 |
| 7.2 | 0.5 | 0 | 100 | 6 |
| 8 | 0.5 | 0 | 100 | 6 |

***Other settings***

|  | |
| --- | --- |
| Sampler T(°C) | 7 |
| Source T(°C) | 150 |
| Dessolvation T(°C) | 350 |
| Capillary voltage (kV) | 2.5 |
| Conce voltage (V) | 41 |
| Desolvation nitrogen gas flow (L/h) | 720 |
| Cone nitrogen gas flow (L/h) | 5 |
| Electrospray ionization mode | Positive |

**Table 4.** Positive Mode Analysis

| **Multi-reaction monitoring (MRM) table (positive mode)** | | | | | | | | |
| --- | --- | --- | --- | --- | --- | --- | --- | --- |
| **CAS** | **Compound** | **MW** | **Formula** | **Retention Time (minute)** | **Transition Window**  **(time)** | **MS/MS Transition (m/z)** | **Cone Voltage (V)** | **Collision Energy (eV)** |
| 99-24-1 | Methylgallate | 184.15 | C8H8O5 | 5.68 | 0.0-8.0 | 184.30>125.01 | 80 | 18 |
| 480-18-2 | Taxifolin | 304.35 | C15H12O7 | 3.22 | 0.0-8.0 | 305.15>152.96 | 256 | 14 |
| 792868-19-0 | Cyanidin-3-arabinoside | 419.4 | C20H19O10+ | 2.19 | 1.0-3.5 | 419.39>287.02 | 46 | 20 |
| 788153-92-4. | Peonidin-3-arabinoside | 433.4 | C21H21O10+ | 3.29 | 2.0-5.0 | 433.11>301.20 | 35 | 20 |
| 324533-67-7 | Delphinidin-3-arabinoside | 435.4 | C20H19O11+ | 1.68 | 0.0-2.5 | 435.35>302.99 | 100 | 18 |
| 749848-37-1 | Petunidin-3-arabinoside | 449.4 | C21H21O11+ | 2.66 | 1.0-4.0 | 449.10>317.00 | 40 | 22 |
| 785047-89-4 | Malvidin-3-arabinoside | 463.4 | C22H23O11+ | 3.69 | 1.0-4.0 | 463.40>331.03 | 100 | 34 |
| 7084-24-4 | Cyanidin-3-glucoside | 449.4 | C21H21O11+ | 1.94 | 0.3-3.0 | 449.39>287.02 | 46 | 20 |
| 68795-37-9 | Peonidin-3-glucoside | 463.4 | C22H23O11+ | 3.04 | 1.5-3.5 | 464.13>301.20 | 35 | 20 |
| 50986-17-9 | Delphinidin-3-glucoside | 465.4 | C21H21O12+ | 1.45 | 0.0-3.0 | 464.89>302.99 | 100 | 18 |
| 6988-81-4 | Petunidin-3-glucoside | 479.4 | C22H23O12+ | 2.36 | 1.2-3.5 | 479.00>317.00 | 40 | 22 |
| 18470-06-9 | Malvidin-3-glucoside | 493.4 | C23H25O12+ | 3.4 | 2.0-4.2 | 492.93>331.03 | 100 | 34 |
| 184466-51-8 | Pelargonidin-3-glucoside | 433.4 | C121H21O10+ | 2.54 | 1.5-4.0 | 433.31>271.14 | 34 | 18 |
| 142506-26-1 | Cyanidin-3-galactoside | 449.4 | C21H21O11+ | 1.69 | 0.3-3.0 | 449.39>287.02 | 46 | 20 |
| 28148-89-2 | Peonidin-3-galactoside | 463.4 | C22H23O11+ | 2.63 | 1.5-3.5 | 464.13>301.20 | 35 | 20 |
|  | Delphinidin-3-galactoside | 465.4 | C21H21O12+ | 1.27 | 0.0-3.0 | 464.89>302.99 | 100 | 18 |
| 28500-02-9 | Petunidin-3-galactoside | 479.4 | C22H23O12+ | 2.07 | 1.2-3.5 | 479.00>317.00 | 40 | 22 |
| 250-055-1 | Malvidin-3-galactoside | 493.4 | C23H25O12+ | 3.07 | 2.0-4.2 | 492.93>331.03 | 100 | 34 |
|  | Pelargonidin-3-rutinoside | 579.5 | C27H31O14+ | 2.99 | 2.0-4.0 | 579.31>271.14 | 46 | 30 |

| **MRM table (positive mode)** | | | | | | | |
| --- | --- | --- | --- | --- | --- | --- | --- |
| **CAS** | **Compound** | **MW** | **Formula** | **Retention Time (minute)** | **Transition Window**  **(time)** | **Ion m/z** | **Cone voltage (V)** |
| 13306-05-3 | Cyanidin | 287.24 | C15H11O6+ | 3.7 | 0.0-8.0 | 287.24 | 25 |
| 134-01-0 | Peonidin | 301.27 | C16H13O6+ | 4.92 | 0.0-8.0 | 301.27 | 25 |
| 8012-95-1 | Delphinidin | 303.24 | C15H11O7+ | 2.56 | 0.0-8.0 | 303.24 | 25 |
| 1429-30-7 | Petunidin | 317.27 | C16H13O7+ | 4.17 | 0.0-8.0 | 317.27 | 25 |
| 10463-84-0 | Malvidin | 331.3 | C17H15O7+ | 5.08 | 0.0-8.0 | 331.3 | 25 |
| 7990-51-9 | Pelargonidin | 271.24 | C15H11O5+ | 4.6 | 0.0-8.0 | 271.26 | 61 |

**Table 5.** Metabolic Parameters in Experimental Mice

| **Characteristics** | **C** | **HF** | **HS** | **HSA** |
| --- | --- | --- | --- | --- |
| Body weight (*g*) | 27.4 ± 0.6 | 45.5 ± 1.1^*^ | 44.5 ± 1.3 | 46.6 ± 1.1 |
| Food intake (*kcal)* | 11.5 ± 0.1 | 16.5 ± 0.2^*^ | 17.6 ± 0.3 | 17.8 ± 1 |
| Blood glucose |  |  |  |  |
| Fasted (m*g/dL*) | 124 ± 5 | 200 ± 10^*^ | 190 ± 10 | 102 ± 13 |
| Non-fasted (m*g/dL*) | 199 ± 9 | 212 ± 6 | 236 ± 14 | 204 ± 6 |
| Body composition |  |  |  |  |
| Fat (%) | 13.2 ± 1.7 | 41.3 ± 0.8^*^ | 44.8 ± 1.2 | 45.6 ± 1.4 |
| Lean (%) | 70.4 ± 1.1 | 54.1 ± 0.7^*^ | 52.1 ± 0.7 | 51.5 ± 1 |
| Water (%) | 13.2 ± 0.2 | 10.8 ± 0.2^*^ | 10.8 ± 0.2 | 11.1 ± 0.2 |

C: Mice fed a standard diet (10% kcal from fat); HF: Mice fed a high-fat diet (60% kcal from fat); HS: mice fed a strawberry-supplemented high-fat diet; HSA: mice fed a strawberry-supplemented high-fat diet and treated with antibiotics cocktail. Values are mean ± SEM; * HF *vs* C, p < 0.05; HS *vs* HF and HSA *vs* HF were non-significant.

**SUPPLEMENTARY FIGURES**

**Figure 1**. Effect of Freeze-Dried Strawberry on Plasma Metabolites

C57BL/6J male mice aged 3 months (n=3) were fasted for 10 h and received freeze-dried strawberry powder (500 mg freeze-dried strawberry powder/kg body weight dissolved in sterile water) *via* oral gavage. The plasma samples were collected and pooled for pilot analyses to identify native strawberry flavonoids (anthocyanins and flavan-3-ols) and their microbial metabolites.

**Figure 2.** Rarefaction and PCoA Plots

Standard Diet ± Strawberry ± Antibiotics


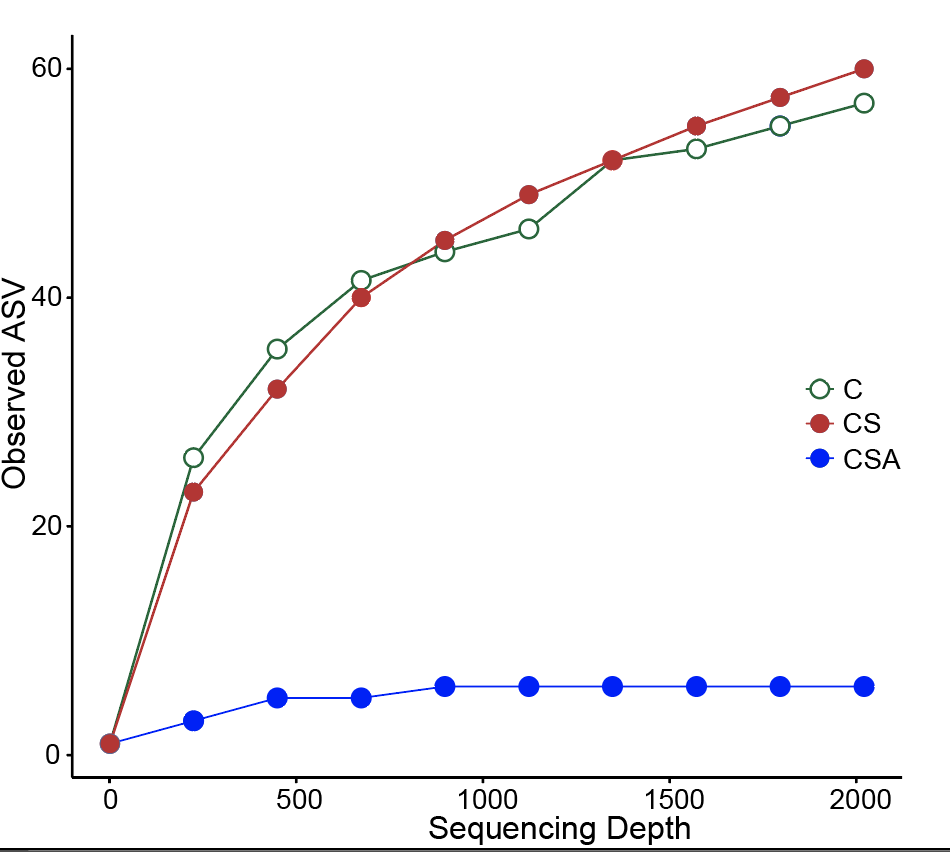


PCoA Plot

Rarefaction Plot

C: Mice fed a standard diet (10% kcal from fat); CS: mice fed a strawberry-supplemented diet; CSA: mice fed a strawberry-supplemented diet and treated with antibiotics cocktail.

High fat Diet ± Strawberry ± Antibiotics


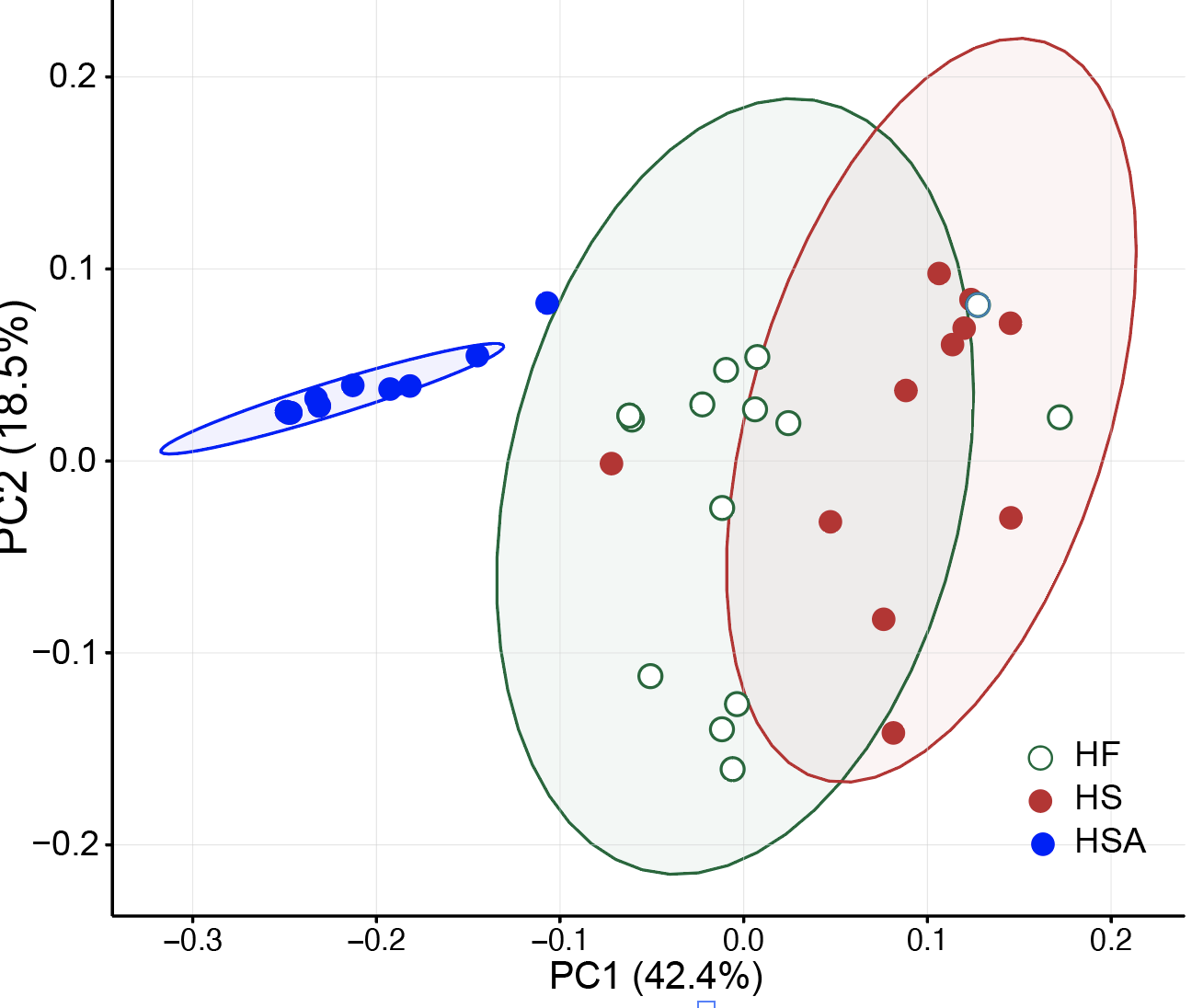

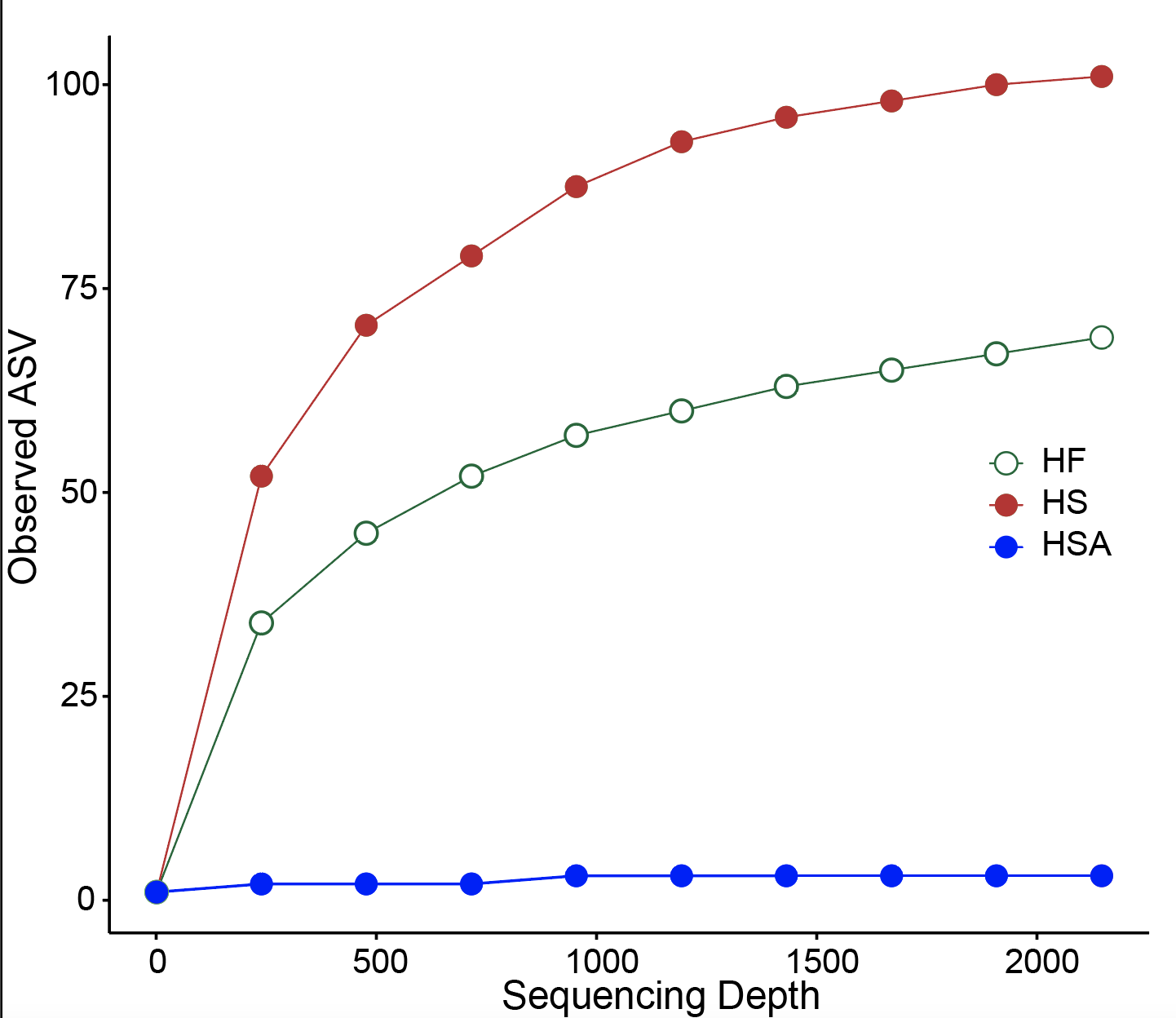


PCoA Plot

Rarefaction Plot

HF: Mice fed a high-fat diet (60% kcal from fat); HS: mice fed a strawberry-supplemented high-fat diet; HSA: mice fed a strawberry-supplemented high-fat diet and treated with antibiotics cocktail.

**Figure 3.** Association Between Gut Microbes, Metabolites and Vascular Inflammation


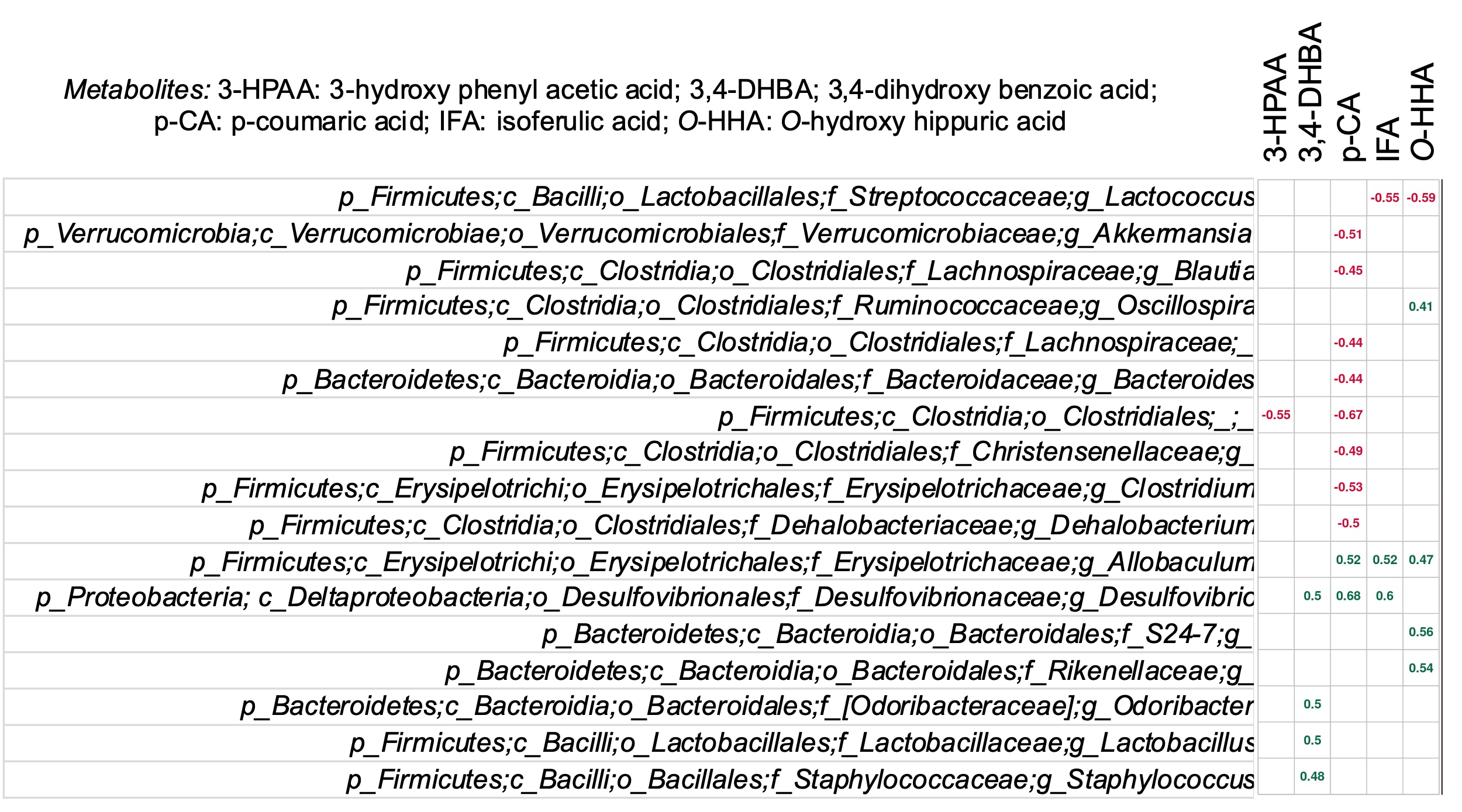


*A. Standard Diet Cohort: Association Between Gut Microbes and Strawberry Metabolites*


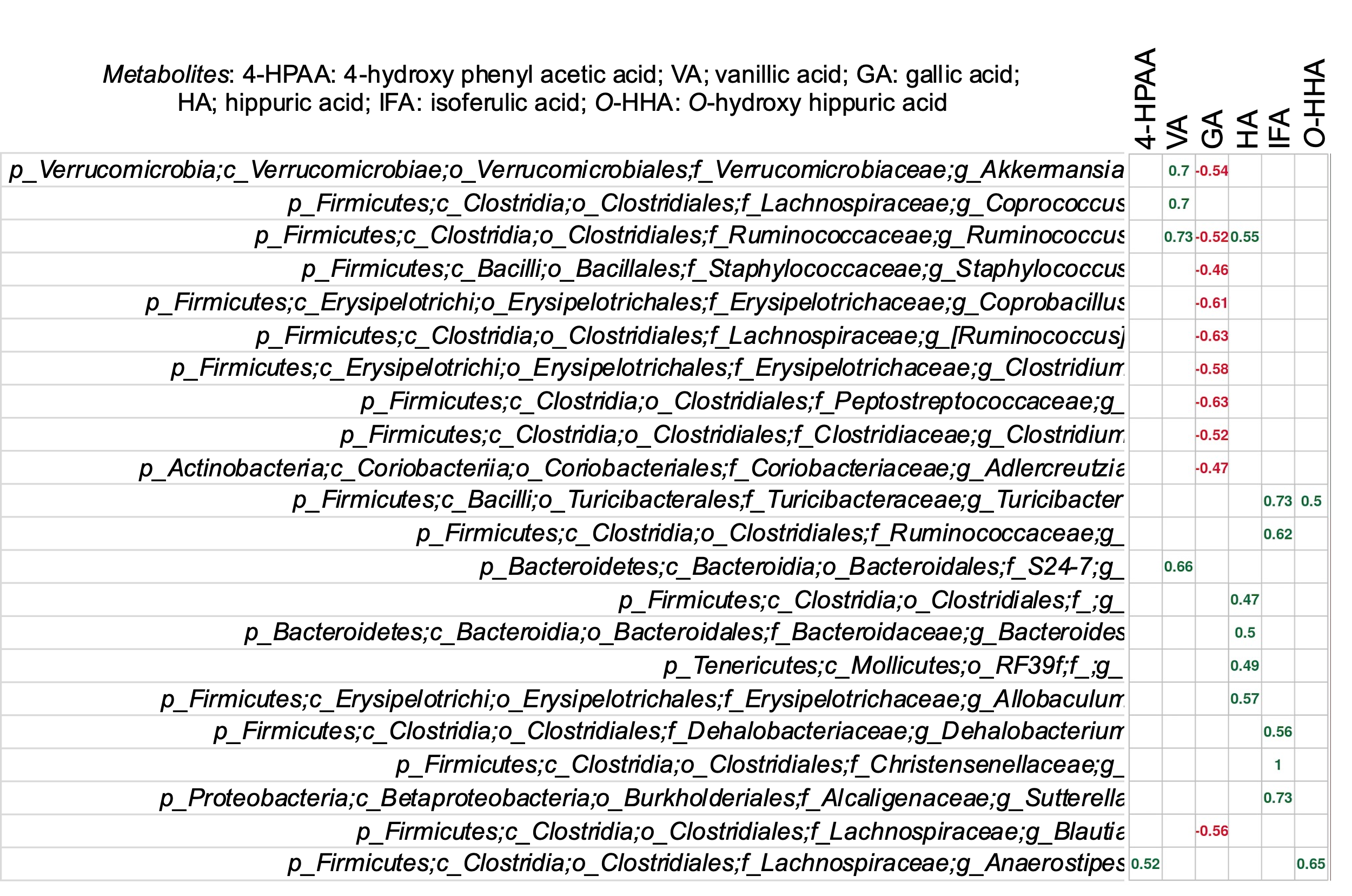


*B. High Fat Diet Cohort: Association Between Gut Microbes and Strawberry Metabolites*


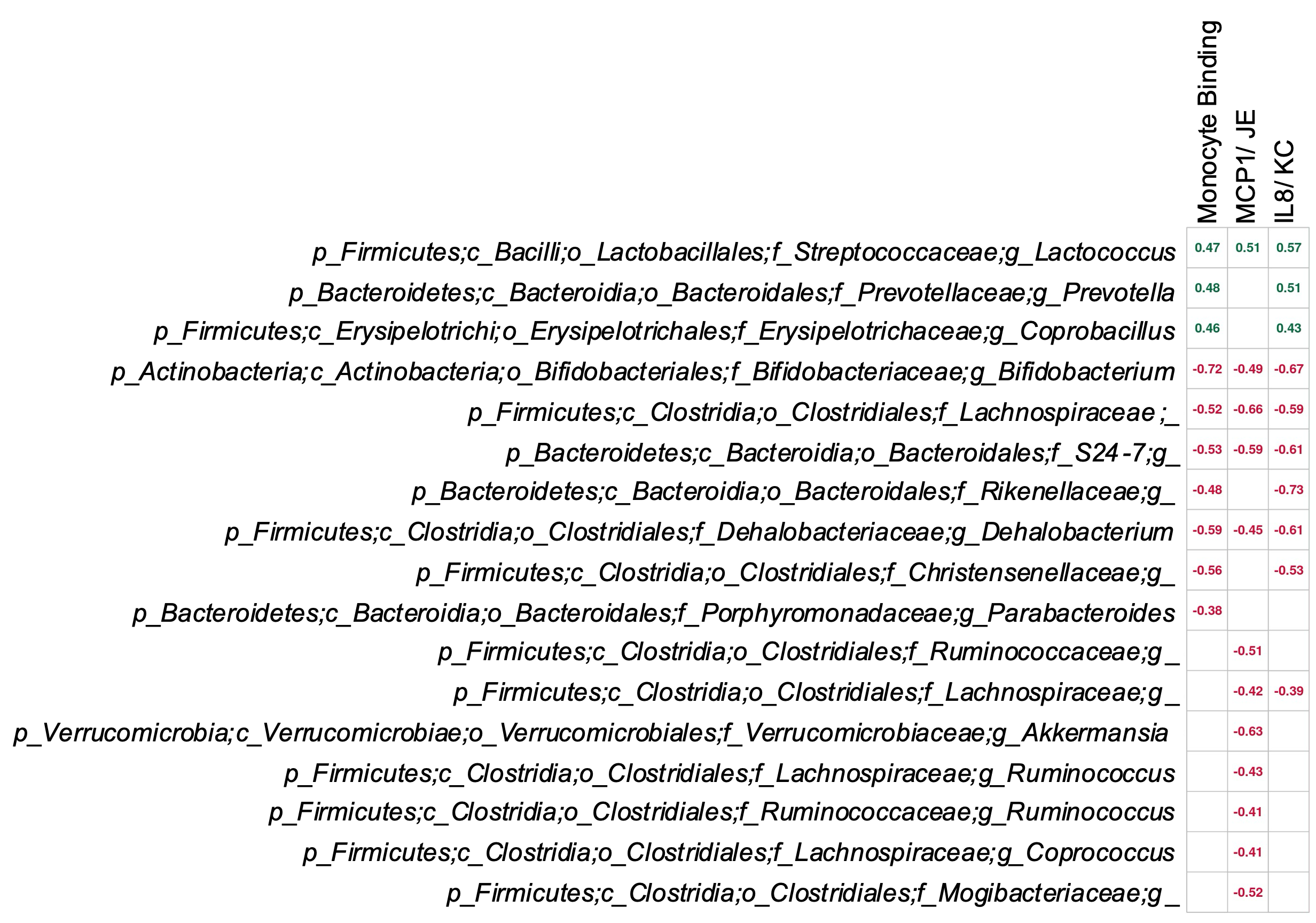


*D. Association Between Gut Microbes and Metabolites*

*C. Association Between Gut Microbes and Indices of Vascular Inflammation*


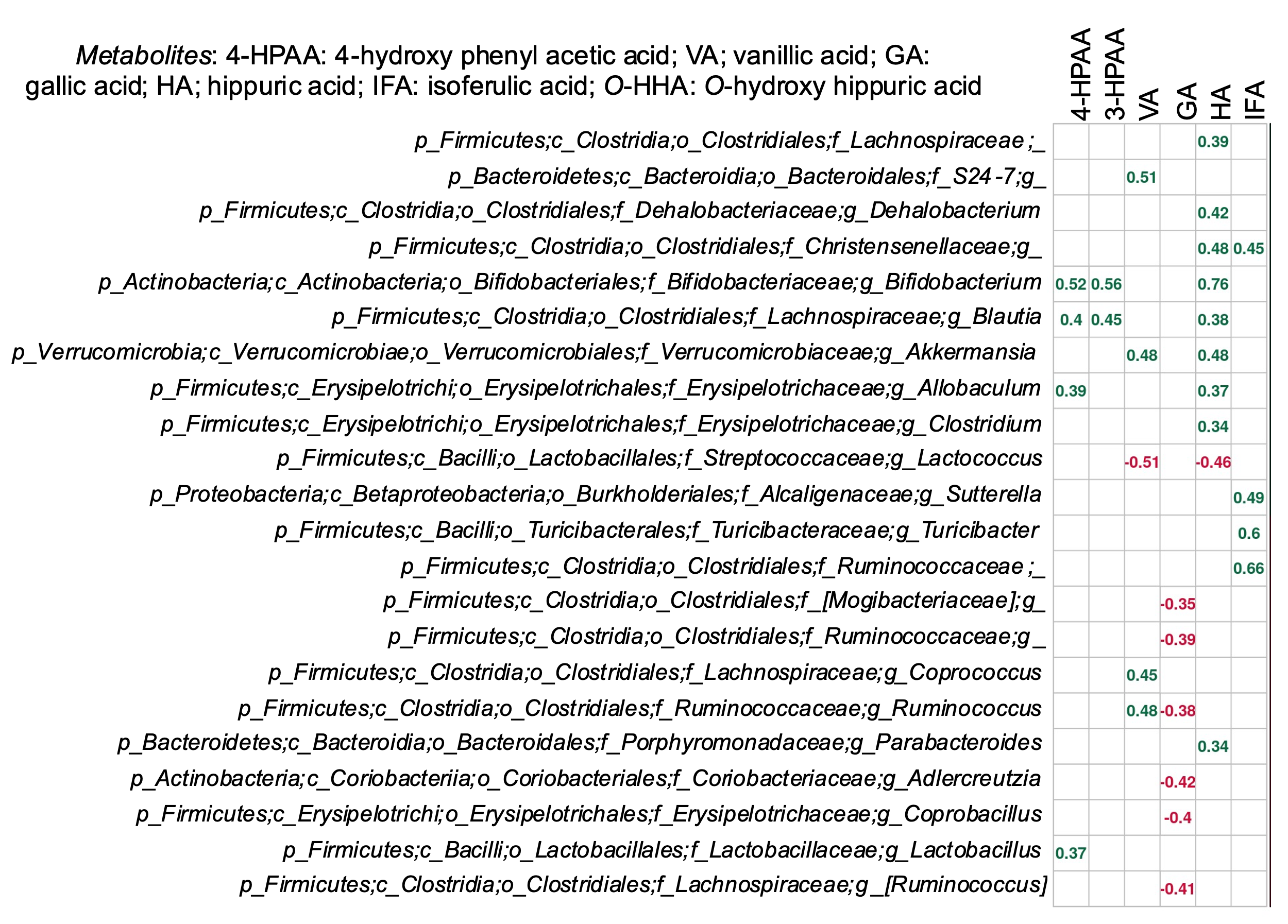


*D. Association Between Gut Microbes and Microbial Metabolites*

*E. Association Between Metabolites and Vascular Inflammation*


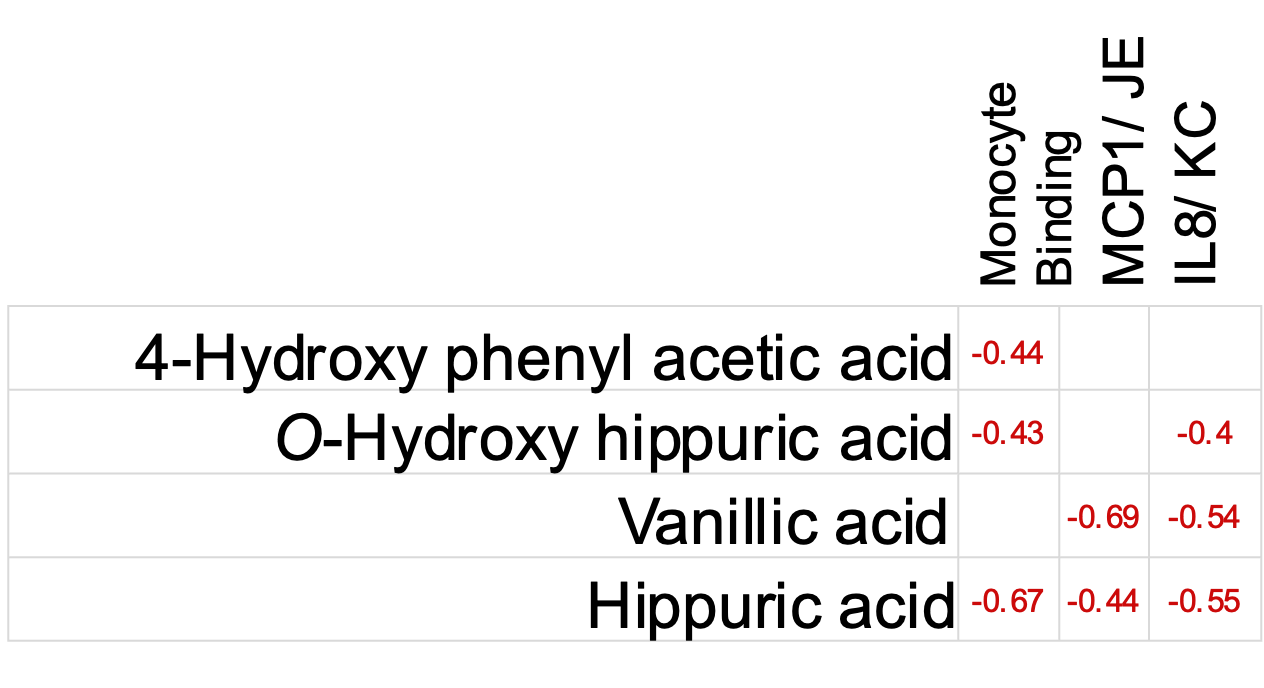


**Figure 4.** Glucose and Insulin Tolerance Tests

Glucose Tolerance Test

*

*Values are mean ± SE (n=8-10);*

** vs C; # vs HF, P < 0.05.*

Insulin Tolerance Test

*

C: Mice fed a standard diet (10% kcal from fat); HF: Mice fed a high-fat diet (60% kcal from fat); HS: mice fed a strawberry-supplemented high-fat diet; HSA: mice fed a strawberry-supplemented high-fat diet and treated with antibiotics cocktail. Values are mean ± SEM; * HF *vs* C, p < 0.05; HS *vs* HF and HSA *vs* HF were non-significant.
